# Association between cortical auditory evoked potentials recorded directly through cochlear implants and post-implantation auditory and speech outcomes

**DOI:** 10.3389/fnins.2025.1701607

**Published:** 2026-01-26

**Authors:** Suhail HabibAllah, Joseph Attias

**Affiliations:** Department for Communication Sciences and Disorders Department, Faculty of Medical Science, University of Haifa, Haifa, Israel

**Keywords:** brain objective measure, cortical auditory evoked potentials, electrical stimulation, intra-cochlear recordings, speech performance

## Abstract

**Introduction:**

Following the demonstration of feasibility in recording electrically evoked cortical auditory evoked potentials (eCAEPs) directly via cochlear implants in both children and adults, the present(CIs), this study aimed to investigate the relationship between eCAEP waveform characteristics and auditory and speech performance among cochlear implant (CI) users. Additionally, the effects of age at implantation and current age on the latencies and amplitudes of the P1, N1, and P2 complex were evaluated, within the framework of developmental auditory cortical plasticity.

**Methods:**

The study included 25 children (mean age 11.5 ± 4 years) and 12 adults; (mean age 33.8 ± 12.3 years), all bilaterally implanted with Advanced Bionics devices. Intracochlear eCAEPs were recorded from 33 implanted ears in children and 21 ears in adults. Recordings were obtained via the contralateral basal electrode (number 13) in response to brief (10 ms) electrical stimulation of the apical electrode of the stimulated CI while recordings were done via basal electrode number 13 of the contralateral recording CI referenced to the CI case. Each recording session lasted approximately 5 minutes. Children ranged in age from 2.7 to 16.7 years (mean: 11.5 ± 4), and adults from 18.5 to 49.1 years (mean: 33.8 ± 12.3). Age at implantation ranged from 0.5 to 8.1 years in children (mean: averaged 1.75 ± 1.4), and from 1.6 to 43.6 years in adults (mean: for children and 23.5 ± 16.6) years for adults. Speech and auditory performance were assessed using CAP, SIR, and monosyllabic word recognition in quiet and noise.

**Results:**

Reliable eCAEPs with all obligatory components were recorded in all ears. Children demonstrated significantly shorter P1-N1 latencies and larger amplitudes compared to the adult cohort in this study. Age at implantation was the strongest predictor of cortical latency, with earlier implantation correlating with shorter P1-N1 latencies. Most children exhibited age-appropriate P1 latencies comparable to normal-hearing peers, whereas only a few adults-those implanted in early childhood-showed similar patterns. In addition, adult responses frequently showed displayed a split P2 waveform, less prominent in children, possibly reflecting altered cortical integration. Notably, significant correlations were found between eCAEP latencies and speech perception scores, particularly in noise, suggesting functional relevance of cortical processing efficiency. Participants with higher CAP and SIR scores exhibited significantly shorter P1-N1 latencies.

**Discussion:**

These findings provide near-field neurophysiological evidence supporting the critical role of sensitive periods in auditory cortical development. The results underscore the value of intracochlear eCAEPs as a neuroscience-based, objective biomarker of auditory cortical function and plasticity in CI users. This approach enables real-time monitoring of neuroplastic changes and offers a novel platform for translational auditory neuroscience in both pediatric and adult implant recipients.

## Introduction

Cochlear implants (CIs) represent one of the most transformative advances in auditory neuroscience and rehabilitation, offering partial restoration of hearing to individuals with severe-to-profound sensorineural hearing loss. Although many recipients achieve meaningful improvements in speech perception and spoken language, outcomes remain highly variable, influenced by a complex interplay of biological, cognitive, and environmental factors (Sharma et al., 2002; Niparko, 2010; Blamey et al., 2013; Holden et al., 2013; Wilson and Dorman, 2008). Notably, early implantation-particularly within sensitive periods of auditory system development-is consistently associated with superior outcomes, reflecting enhanced cortical plasticity during early childhood and a greater capacity for adaptive neurodevelopment (Dettman et al., 2007; Sharma et al., 2005).

One of the central challenges in cochlear implant care and auditory neuroscience is the objective evaluation of central auditory function, especially in pediatric populations or individuals with limited behavioral cooperation. While traditional speech perception measures and clinician-rated scales, such as the Categories of Auditory Performance (CAP) and Speech Intelligibility Rating (SIR), provide valuable clinical insights, they rely on behavioral input and may not capture the full extent of underlying neural activity (Archbold et al., 1998; Gifford et al., 2008).

In this context, cortical auditory evoked potentials (CAEPs) have gained prominence as noninvasive neural markers of auditory cortical processing. The P1-N1-P2 complex, which constitutes the most commonly studied CAEP components, reflects successive stages of cortical encoding of auditory stimuli, originating in primary and secondary auditory cortices, such as Heschl's gyrus and planum temporale (Ponton et al., 2000; Edwards et al., 2009). These evoked responses represent the brain's ability to process the temporal and spectral structure of sound and are modulated by maturation, sensory experience, and higher-order auditory networks. Notably, the Acoustic Change Complex (ACC), a subtype of CAEP elicited by within-stimulus contrasts, has been associated with speech-in-noise perception, a crucial function for real-world auditory comprehension (Martin and Boothroyd, 1999; He et al., 2015).

A growing body of literature has established CAEPs as robust biomarkers of cortical plasticity and auditory development in both pediatric and adult CI users. In children, longitudinal studies demonstrate that a progressive reduction in P1 latency following implantation tracks the maturation of auditory cortical networks and correlates with improvements in speech and language abilities (Sharma et al., 2005, 2025; Silva et al., 2017; Atilgan et al., 2023). For example, Guo et al. (2016) found that consistent CAEP presence predicted better Mandarin Early Speech Perception (MESP) outcomes over a 4-year period, and similar associations were shown by Xiong et al. (2022) and Wang et al. (2023) using IT-MAIS, LittlEARS, CAP, and SIR. Silva et al. (2017) tracked CAEP maturation in children with cochlear implants and found a significant reduction in P1 latency at nine months post-activation. Crucially, shorter P1 latencies correlated with improved scores on IT-MAIS/MAIS and MUSS and higher levels of auditory discrimination, reinforcing P1 as an objective neurophysiological indicator of functional auditory development. Atilgan et al. (2023) demonstrated that children with sequential bilateral cochlear implants show normal auditory maturation, although the second implant's cortical response latency takes up to a year to match the first. Their findings suggest that the timing of the first implant's experience influences the second ear's rate of compensation.

In adults, scalp recorded CAEPs have also demonstrated sensitivity to speech perception abilities. CI users with superior performance tend to exhibit P1–N1–P2 responses that more closely resemble those of normal-hearing individuals, while those with poorer outcomes show delayed or reduced cortical responses, especially in the P2 component (Groenen et al., 2001; Kelly et al., 2005). Importantly, the integrity of the N1–P2 complex in noisy listening conditions correlates with real-world speech-in-noise understanding (Anderson et al., 2012). These findings highlight the potential of CAEPs as translational tools bridging cortical neurophysiology and auditory behavior.

More recently, electrically evoked CAEPs (eCAEPs), recorded from scalp electrodes in response to direct CI stimulation, have demonstrated potential for clinical application in implant verification and longitudinal monitoring (Tavora-Vieira and Ffoulkes, 2023; Bogdanov et al., 2024; Liebscher et al., 2018). These responses offer insight into how cortical circuits respond to electrical stimuli, contributing to our understanding of cortical adaptation following sensory prosthesis.

While scalp-recorded CAEPs have demonstrated clinical potential, they require external hardware, are susceptible to motion and electrical noise, and often demand controlled environments-limitations that reduce their utility in everyday clinical settings. To overcome these constraints, a novel technique has emerged: intracochlear recording of eCAEPs directly through the CI electrodes. This method leverages the existing implanted system to capture cortical responses without the need for scalp electrodes, resulting in superior signal-to-noise ratios and reduced testing time-features especially valuable for infants and young children (Attias et al., 2022; Bell-Souder et al., 2024; HabibAllah et al., 2025). Moreover, intracortical recordings have shown that CI stimulation can elicit phase-locked cortical activity to speech-like stimuli, supporting the physiological relevance of these responses to speech perception (Nourski et al., 2013; Aldag et al., 2022).

While feasibility of the intra cochlear eCAEPs has been established, the functional and clinical significance of these implant-recorded eCAEPs remains underexplored. Specifically, the relationship between eCAEP waveform characteristics and auditory or speech perception outcomes has yet to be systematically examined.

In this study, we investigated the association between intracochlear eCAEPs and behavioral measures of speech and auditory performance in pediatric and adult CI users. By aligning these objective neural markers with functional outcomes, we aim to validate eCAEPs as neuroscience-based indicators of cortical auditory processing and plasticity. This research offers a promising step toward integrating direct neural monitoring into individualized CI rehabilitation and developing neuroscience-informed frameworks for clinical decision-making in auditory prosthetics.

## Materials and methods

### Participants and ethics declarations

In this study 37 subjects (54 ears), children and adults aged from 2.5 to 50 years (17 females and 20 males) participated. All were bilateral AB CI users. All children had congenital severe to profound hearing loss and were implanted in both ears at young ages ranging from 6 months to 8.1 years from birth. All the adults had a congenitally severe to profound hearing loss and mostly were post-lingual implanted sequentially during adulthood period. Table 1 details the demographic data for the pediatric, adult and all participants including, gender, etiology, current age, age of implantation, sequential or simultaneous and pre or post lingual implantation. Subjects or their parents provided written informed consent to participate in the study, but they were free to stop their participation at any point during the study. The protocol of the study was approved by the Investigational Review Board (IRB: 0617-19-RMC, Schneider Children Medical Center, Israel) and all methods were performed in accordance with the relevant guidelines and regulations. The current mean age of the CI children group was 11.09 ± 4.1 yrs with a minimum of 2.5 yrs and maximum of 16.7 yrs. The mean implantation age was 2.3 ± 2.3 yrs ranging from 0.6 to 8.7 post-natal years. All had congenital deafness with etiologies including genetic (11) Auditory Neuropathy (2), LVA (1), CMV (1), and the rest with unknown source (5). Prelingual deafness was defined as onset of hearing loss or implantation before approximately 3 years of age, corresponding to the critical period for early language acquisition (Chen et al., 2024).

**Table 1:** Demographic data for the pediatric, adult and all participants including, gender, etiology, current age, age of implantation, sequential or simultaneous and pre or post lingual implantation.

**Demographic and cochlear implant data**	**Pediatric**	**Adults**	**Total**
*N* (subjects)	25	12	37
*N* (tested ears)	33	21	54
M/F	13/12	7/5	20/17
**Etiology**			
Genetic	10	4	14
ANSD	3	1	4
ASD	1		1
GDD	1		1
CMV	1		1
LVA	1		1
Usher		2	2
Unknown	8	5	13
**Current age (years)**			
Min	2.7	18.5	
Max	16.7	49.1	
Mean ± SD	11.5 ± 4	33.8 ± 12.3	
**Age at cochlear implantation (years)**			
Min	0.5	1.6	
Max	6.5	43.6	
Mean ± SD	1.74 ± 1.4	23.5 ± 16.6	
Simultaneous CIs (ears)	31	2	34/20
Sequential CIs (ears)	3	19	
Pre lingual deafness	30	4	34/20
Post lingual deafness	3	17	

ANSD, auditory neuropathy spectrum disorder; ASD, autism spectrum disorder; GDD, global development delay; CMV, cytomegalovirus; LVA, large vestibular aqueduct.

### Research set-up

In the current study, bilaterally implanted cochlear implant (CI) systems were used such that one device functioned as the electrical stimulator, while the contralateral device was dedicated to recording eCAEPs. Specifically, we used the Advanced Bionics (AB) system, which included the AB Clinical Programming Interface (CPI-3). The CPI-3 was connected to two Naída CI Q90 speech processors. Each speech processor was linked to an RF cable and headpiece for wireless communication with the implanted device in each ear for forward and backward telemetry.

Both speech processors were also connected to a trigger dongle to allow the transmission and reception of input and output trigger events. The speech processor on the “stimulating” (ipsilateral) side delivered biphasic electrical stimuli and simultaneously generated a trigger signal at the onset of each stimulation frame. This trigger was sent to the contralateral processor. This setup enabled synchronized stimulation and recording eCAEPs from two sources simultaneously. Hence, all eCAEPs reported in this study were collected directly via the contralateral, non-stimulated CI. Explanation regarding this will be explained later in this section.

The electrical stimulus consisted of a biphasic pulse train of 10 ms total duration, with cathodic-first polarity. The default stimulation phase width was 36 μs. The pulse rate used was 500 pulse per second. This value was fixed across all participants unless excessive electrode impedance at the stimulation site required increasing the pulse width to maintain electrical compliance. The number of sweeps per trial was set to 150, which corresponded to approximately 4 min per experiment trial. The presentation level was set individually for each subject according to their most comfortable level of stimulation, i.e., the participant feedback at a “loud but comfortable” level, and in participants who could not provide behavioral feedback, levels were set according to the participant's daily program map (M-level) at the stimulated electrode.

The experimental setup and parameters were controlled using Advanced Bionics' proprietary Bionics Ear Evoked Potential (BEEP) research app (version 1.0.1.1). The BEEP app enabled control of all stimulation parameters, managed communication between the two speech processors, and coordinated trigger signals. This software allowed eCAEPs to be directly measured via the intracochlear electrode on the contralateral side without the need to concatenate short segments of recording windows, as was required in earlier studies to record eCAEPs.

**Figure 1:**
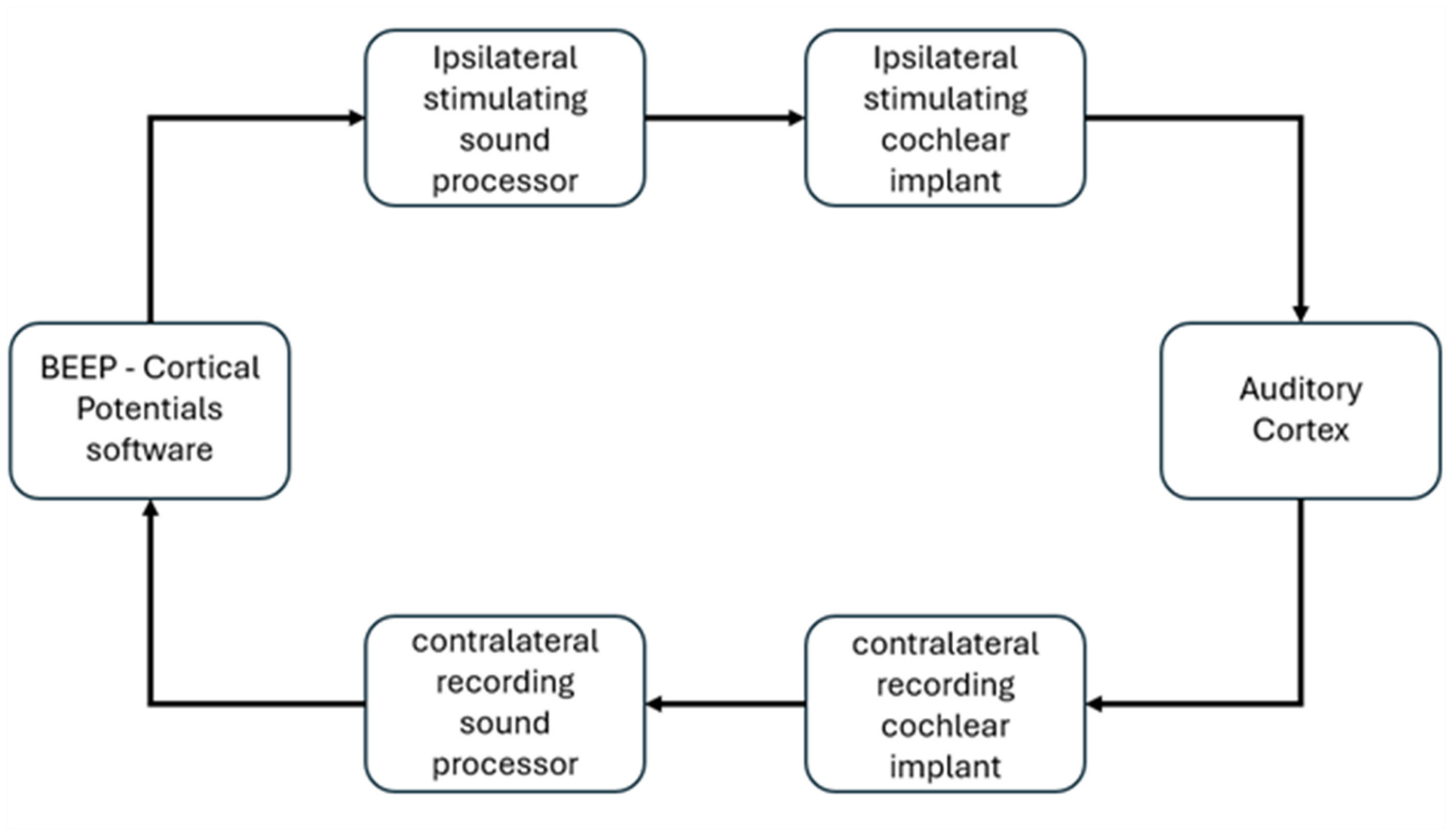
Shows the set up for Bilateral eCAEP test configuration. The arrows indicate the flow direction of the signals or data going in or out of software.

Stimulation was delivered to the most apical electrode labeled as #1 (by the manufacturer AB) on the ipsilateral CI, i.e., the stimulating side, and recordings were collected from basal electrode labeled as #13 on the contralateral CI, i.e., the recording side. Electrode #13 was selected based on its relatively low impedance, although electrodes 12 or 14 were used if electrode 13 was unavailable due to high impedance or other reasons (disconnected electrode). The apical electrode was chosen for stimulation to engage low-frequency pathways critical for speech envelope processing. The reference electrode for stimulation was the implant housing (case ground), and the recording reference was the same. This electrode configuration follows the protocol described in prior studies of acoustically evoked CAEPs (Attias et al., 2022). We elected to maintain this setup because it reliably yielded strong and interpretable cortical responses. Intracochlear eCAEPs were transmitted back to the BEEP app via RF backward telemetry for visualization and signal processing. Figure 1 shows the set-up of the test environment. For detailed visualization of the set-up, the reader is referred to HabibAllah et al. (2025).

All testing took place in a sound-attenuated booth with participants seated in a recliner chair. Participants or their guardians were fully briefed on the testing procedure prior to data collection. Adult and teenage participants were instructed to silently count the number of stimuli presented to help maintain attention. Younger children (under 6 years) were shown a silent video on a tablet to help them remain quiet and minimize movement and blinking artifacts. Participants were allowed short breaks between recording sessions as needed. The implanted ear selection for the eCAEP recording was based on electrode impedance and recording quality. When both ears met impedance criteria and time allowed, recordings were obtained bilaterally. In cases where only one ear could be recorded (due to young age, limited cooperation, or time constraints), we consistently selected the ear implanted first, as it typically reflects longer auditory experience and more stable cortical adaptation. This approach ensured consistency and minimized variability related to inter-implant delay. Pediatric and adult cohorts were analyzed separately for descriptive purposes to illustrate developmental differences, while all inferential analyses were conducted using continuous variables such as age at implantation.

### Data collection configuration

**Figure 2:**
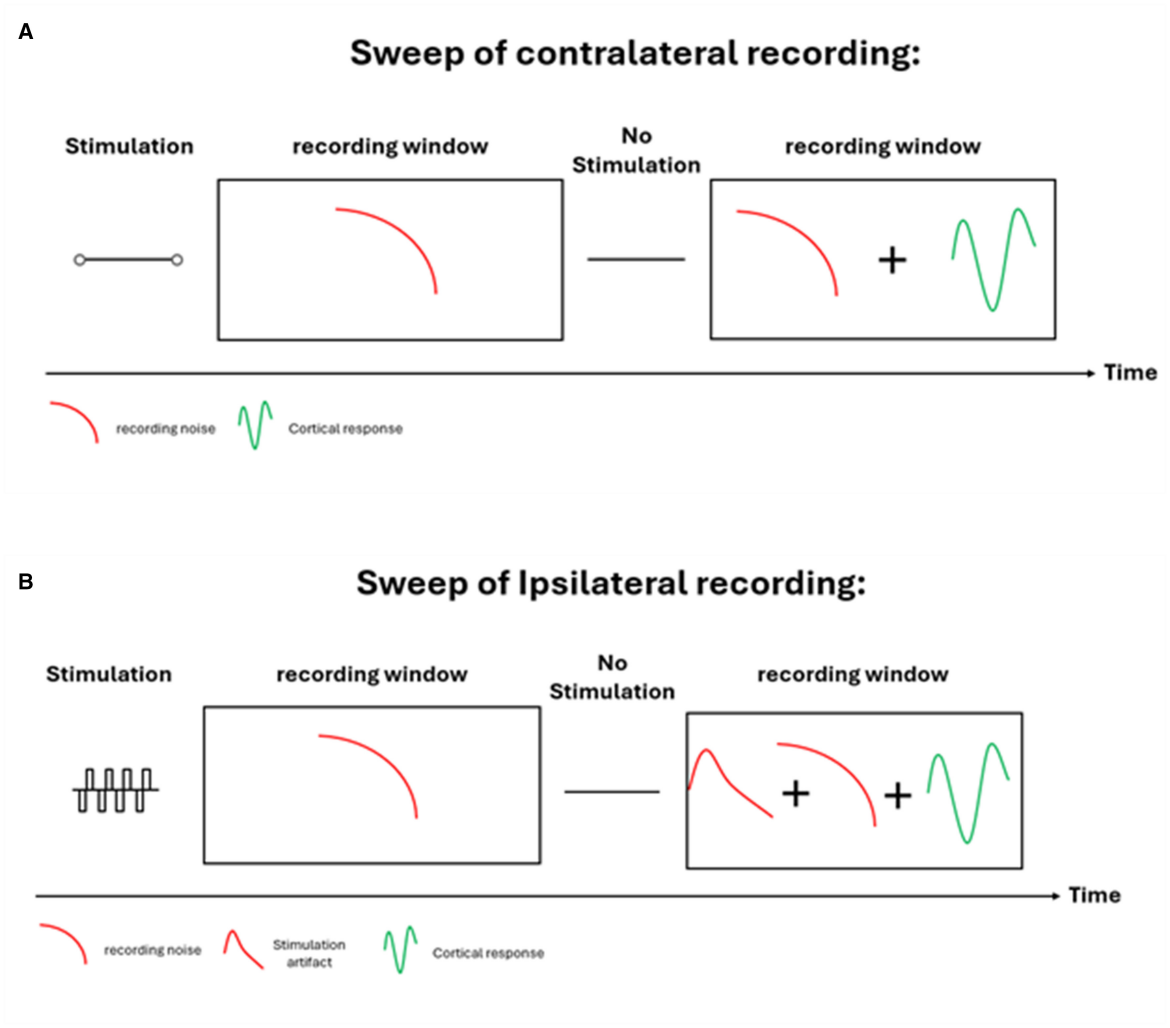
Illustration of the Sweep structure in each experimental trail. The upper panel **(A)** shows the contralateral sweep recording, and the lower panel **(B)** shows the ipsilateral sweep recording.

As stated earlier, eCAEP were collected using contralateral configuration, i.e., the cortical response was collected from the side with no stimulation delivered. Figure 2 shows the structure of the sweep in each recording. eCAEP measurement sweep consisted of two sequential parts. The first part was the stimulation at MCL (most comfortable level) condition, which involved a 10 ms stimulation period, followed by a 500 ms recording window. The second part, the “no stimulus” condition, which involved a 10 ms segment with zero-amplitude stimulation (quiet), followed by a 500 ms recording window. For each sweep, thus, the total time was on average about ~1.02 s. The stimulus was presented every ~1.02 s, i.e., the stimulus repetition rate was about ~0.9 Hz. Each CI eCAEP session comprised 150 sweeps.

As seen in Figure 2, the recording of eCAEP can be established using two configurations. The upper panel illustrates the contralateral configuration, and the lower panel illustrates the Ipsilateral configuration. In both configurations, one limitation is encountered when recording cortical responses directly through the CI system. The recording artifact, which reflects the behavior of the electrical recording circuitry. This artifact is present as soon as the recording window is initiated and appears under all conditions-including ipsilateral and contralateral CIs-and during “no stimulus” zero-amplitude stage and the MCL high-amplitude stimulation stage. This artifact is a footprint of the internal implant system, and it is related to the switching behavior of the circuit to start recording, including the amplifier, the Digital to Analog Converter (DAC) and other electrical components of the implant. To manage this issue, a subtraction technique was applied, where the “no stimulus” recording was subtracted from the MCL recording. In the “no stimulus” stage, which is 10 ms of zero-amplitude stimulation, no cortical response or stimulation artifact is expected. The resulting data reflects only the intrinsic behavior of the recording circuitry for that particular trial. By contrast, during the MCL stage, which 10 ms of stimulation at the most comfortable loudness level, the recording contains two components: the true cortical evoked response and the recording-circuit artifact. For the ipsilateral configuration it will also include the stimulation artifact. Subtracting the “no stimulus” condition from the MCL condition eliminates the circuit artifact common to both, leaving only the cortical response and stimulation artifact.

The second limitation is the electrical stimulation artifact. Higher stimulation amplitudes produce larger artifacts. To minimize or eliminate this artifact, the cortical responses were collected and analyzed only on the contralateral (non-stimulated) CI side, while data from the ipsilateral side were disregarded, as it contained the stimulation artifact. This strategy, combined with the “no stimulus”—MCL subtraction technique, yielded a cleaner signal. Specifically, while recording ipsilaterally after stimulation, the artifact amplitude can be significant enough to obscure the evoked response especially at higher stimulation levels, and its presence can be seen for 300 ms in the recording window. In contrast, recording on the contralateral side, i.e., stimulating one ear and recording from the other ear as shown in the upper panel of Figure 2, essentially eliminates the stimulation artifact because the electrical artifact is not transmitted across to the opposite intracochlear electrode. This dual approach ensured that the data reflected only the auditory-evoked cortical response and the minimal recording-circuit artifact after subtraction. Based on the above reasoning, subtracting the “no stimulus” stage from the MCL stage eliminates the recording artifact, leaving only the cortical evoked response. To achieve this, all subjects in this study were bilaterally implanted, allowing one cochlear implant to provide stimulation while the contralateral, non-stimulated implant was used exclusively for recording. This setup ensured that stimulation artifacts did not contaminate the contralateral recordings.

The eCAEP recordings in this study were obtained intracochlearly via the cochlear implant electrodes, rather than from scalp electrodes. Therefore, common muscle artifacts such as eye blinks or facial movements, which typically affect scalp-recorded CAEPs, do not occur in this recording configuration.

### Data analysis

Data was post-processed using a custom-built MATLAB application. Each raw data set contained 150 sweeps per condition. The “'no stimulus” sweeps were then subtracted from the MCL sweeps before averaging. Following subtraction and averaging, the linear trend was removed, and the resulting waveform was bandpass filtered (1–20 Hz) using a second-order infinite-impulse-response (IIR) filter. Finally, after averaging 150 sweeps, a moving average smoothing algorithm with a 33 ms window was applied to improve signal-to-noise ratio prior to visual and quantitative analyses.

Following offline analysis, co-author JA-blinded to the recording method (cochlear implant vs. scalp)-manually marked the P1, N1, P2, and N2 peak latencies. Peak identification was guided by previously published latencies for scalp-recorded eCAEPs in normal-hearing individuals and pediatric cochlear-implant recipients. Specifically, for pediatric eCAEPs, P1 was defined as the most prominent positive peak between 55 ms and the subsequent largest negative peak (N1) occurring within 200 ms after stimulus onset. P2 was defined as the largest positive peak following N1, occurring before approximately 280 ms, and N2 was defined as the subsequent negative peak after P2, appearing within 370 ms of the stimulus. Peak-to-peak amplitudes were calculated for the P1–N1, N1–P2, and P2–N2 components to assess the relative magnitude of each response.

### Tests of auditory and speech intelligibility

In four children, speech perception testing could not be conducted due to specific limiting factors: young age, young age combined with a diagnosis of ASD, auditory neuropathy spectrum disorder (ANSD) with associated psychomotor delay, and global developmental delay. All remaining participants completed speech in quiet and speech-in-noise test to evaluate auditory speech perception under controlled conditions. The test was conducted in a calibrated, sound-attenuated anechoic chamber to minimize any extraneous noise and reverberations. Speech stimuli consisted of phonetically balanced (PB) word lists presented at MCL level (on average 55 dB HL), and the background noise was presented 10 dB below that MCL, ensuring a fixed SNR of 10 dB for all subjects. The language of the word list was adapted to the participant's native language (Arabic or Hebrew). The word lists were adapted to each participant's native language (Arabic or Hebrew). Both PB lists are validated and widely used in clinical practice and as a standard in the country. Furthermore, the lists were administered by an audiologist who is a native speaker of the respective language. For the speech intelligibility tests in noise, both speech and noise stimuli were presented simultaneously from a single loudspeaker positioned at 0° azimuth directly in front of the participant at one meter distance.

Each participant was initially presented with a 25-word PB list. If they incorrectly repeated more than three words, an additional 25-word PB list was administered to allow more accurate measurement. Error weighting was calculated separately for one-list and two-list presentations, and scores were expressed as the percentage of correct responses. Speech perception testing was also performed using the same implant that served as the stimulating device during the eCAEP recordings. For participants tested on both ears, the stimulating side was switched accordingly. During testing, the participant listened to words presented through a front-facing loudspeaker, both quietly and in noise.

Each listening condition was tested in quiet (speech-only presentation) as well as in noise (+10 dB SNR). This allowed a direct comparison of speech perception under different auditory configurations across quiet and noisy environments.

Functional auditory performance was assessed using the Categories of Auditory Performance (CAP) scale as described by Archbold et al. (1998). The CAP is an 8-point hierarchical scale that ranges from 0 (“No awareness of environmental sound”) to 7 (“Use of the telephone with a known speaker”). CAP scoring was conducted on the day of the electrophysiological CAEP recordings by the research team. Assessments were based on: structured observations during clinical sessions; review of participant performance across daily activities; reports from parents or caregivers, following established CAP guidelines. For each participant, the highest CAP level that was observed reliably in day-to-day communication was assigned as the CAP score.

Speech intelligibility was evaluated using the Speech Intelligibility Rating (SIR) scale proposed by Allen et al. (2001). The SIR is a five-point ordinal scale ranging from 1 (“Connected speech is unintelligible”) to 5 (“Connected speech is easily understood by all listeners in everyday contexts”). Ratings were conducted during the same session as the eCAEP testing in the anechoic chamber.

Speech perception tests were administered in the participant's native language (Arabic or Hebrew) using phonetically balanced (PB) word lists that are standard clinical tools in Israel and have been previously validated for phonetic balance and comparability. The audiologist conducting the test was a native speaker of the same language as the participant, ensuring accurate administration and scoring.

All hearing and language assessments were conducted by experienced audiologists who were blinded to brain recordings and were not involved in their acquisition.

## Results

The study enrolled 25 children and 12 adults with bilateral AB implants, from whom eCAEP recordings were obtained from a total of 54 ears. In children, recordings were obtained from both implanted ears in eight participants and from one ear in 17 participants (33 ears in total). In adults, eCAEP recordings were obtained from both implanted ears in nine participants and from one ear in three participants (21 ears in total).

As detailed in Table 1, genetic (*n* = 11) and congenital (*n* = 14) etiologies predominated in pediatric hearing loss, whereas idiopathic causes were most prevalent among adults (*n* = 13). Age at test ranges spanned 2.7–16.7 years for children and 18.5–49.1 years for adults, with implantation ages varying between 0.5–6.5 years (pediatric) and 1.6–43.6 years (adult). Gender distribution showed 17 males and 16 females in the pediatric cohort, compared to 13 males and eight females in adults. The vast majority of pediatric participants (*n* = 31 ears) underwent simultaneous bilateral implantation, with only one child a sequential CI. The vast majority of pediatric implantations (30 ears) occurred during the prelingual period, while three ears were implanted postlingually. Among adults, four ears were implanted during the prelingual period and 17 ears postlingually. The current ages of the adults who had one ear implanted during the prelingual period are 18.5, 18.8, 20.1, and 21.1 years.

**Figure 3:**
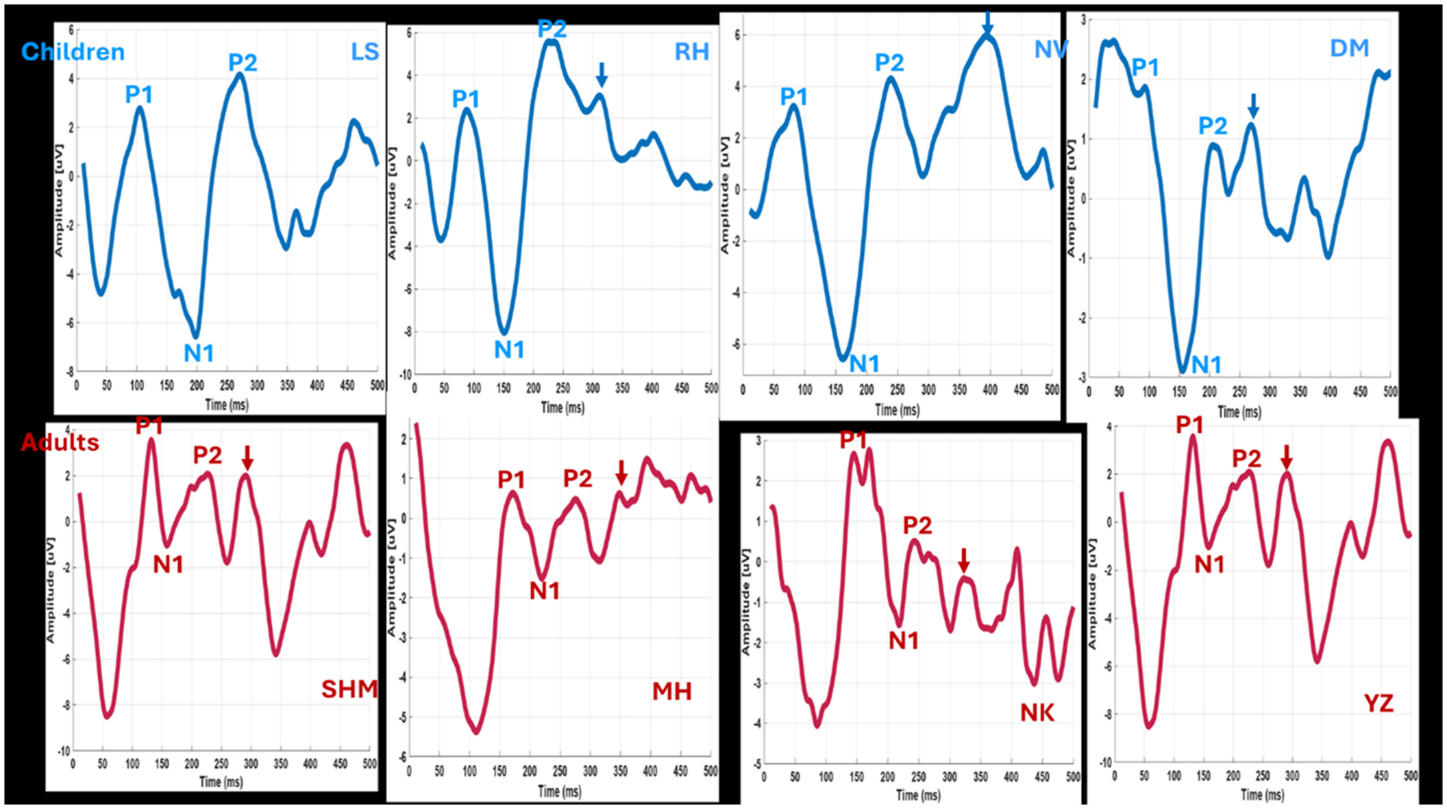
Individual eCAEP patterns and variability in four children and four adults. Note the split in P2 peaks across the subjects.

eCAEP recordings were successfully obtained from all the ears of all study participants. Figure 3 illustrates the individual variability in recording patterns among four children and four adults. The P1, N1, and P2 components were identified and marked for each participant. It was observed that the latencies of the P1–N1 components in children were earlier, and their amplitudes were higher compared to those recorded in adults. Additionally, in both groups, the P1 and N1 waves typically consisted of a single peak, while the P2 wave often exhibited two consecutive peaks (split, indicated by arrows). This splitting of the P2 component was more pronounced in adults.

**Figure 4:**
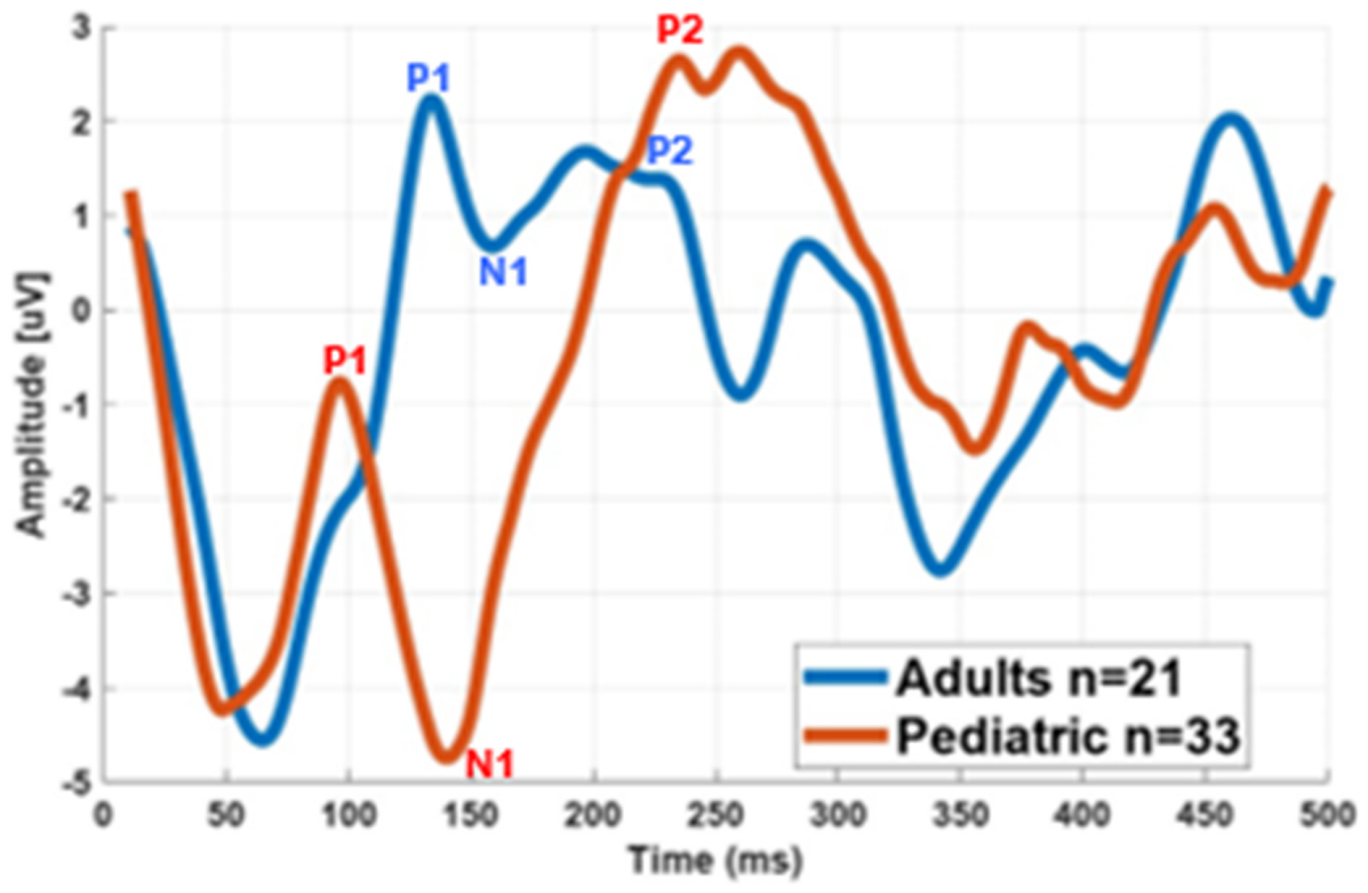
Grand average eCAEP waveforms for children's and adults' implanted ears. The P1–N1 peaks occur earlier with greater amplitudes in children compared to adults. Note the P2 split more pronounced in adults than children.

Figure 4 illustrates the average eCAEP recordings for the entire group of all children and adults. The average eCAEP pattern in children shows earlier and larger P1–N1 latencies compared to adults. Additionally, the P2 component appears as two consecutive peaks (indicated by arrows), which are more pronounced in adults than in children.

Descriptive statistics (median and interquartile range), Kruskal–Wallis *H*-statistics, and *p*-values for latency and amplitude measures are summarized in Table 2. Statistical analysis revealed that children exhibited significantly shorter LP1 and LN1 latencies and larger amplitudes compared to adults, while P2 latency did not differ significantly between groups. Latency measures (LP1, LN1, and LP2) are expressed in milliseconds (ms), and amplitude measures (AP1, AN1, and AP2) in microvolts (μV). Notably, P1–N1 peak amplitudes in children reached up to 20 μV, whereas the maximum amplitude recorded in adults was 13 μV. Among the pediatric cohort, the shortest observed P1 latency was 71 ms and the longest was 147 ms. This group included three children with unique auditory histories: two were bilaterally implanted at a very young age but raised in a signing environment, and one received a second implant more than 4 years after the first. These cases highlight variability in cortical auditory responses despite early implantation. Overall, the findings confirm that earlier cortical responses and larger amplitudes characterize the pediatric group compared to adults.

**Table 2:** Descriptive statistics (Median, IQR), H-statistics, and *p*-values for latency and amplitude measures.

**Component**	**Group comparison**	**Median children**	**Median adults**	**IQR**	**H-statistic**	***p*-value**
LP1	Children vs. adults	95 ms	140 ms	88–104/128–162	18.6	< 0.001
LN1	Children vs. adults	150 ms	185 ms	140–160/170–210	15.2	< 0.001
LP2	Children vs adults	240 ms	245 ms	220–260/230–270	2.1	N.S
AP1	Children vs. adults	7.0 μV	4.0 μV	5.0–9.5/2.5–6.0	7.8	0.007
AN1	Children vs adults	8.0 μV	2.5 μV	6.0–10.0/1.5–4.0	16.4	< 0.001
AP2	Children vs. adults	5.5 μV	3.8 μV	4.0–7.0/2.5–5.0	5.3	0.02

**Figure 5:**
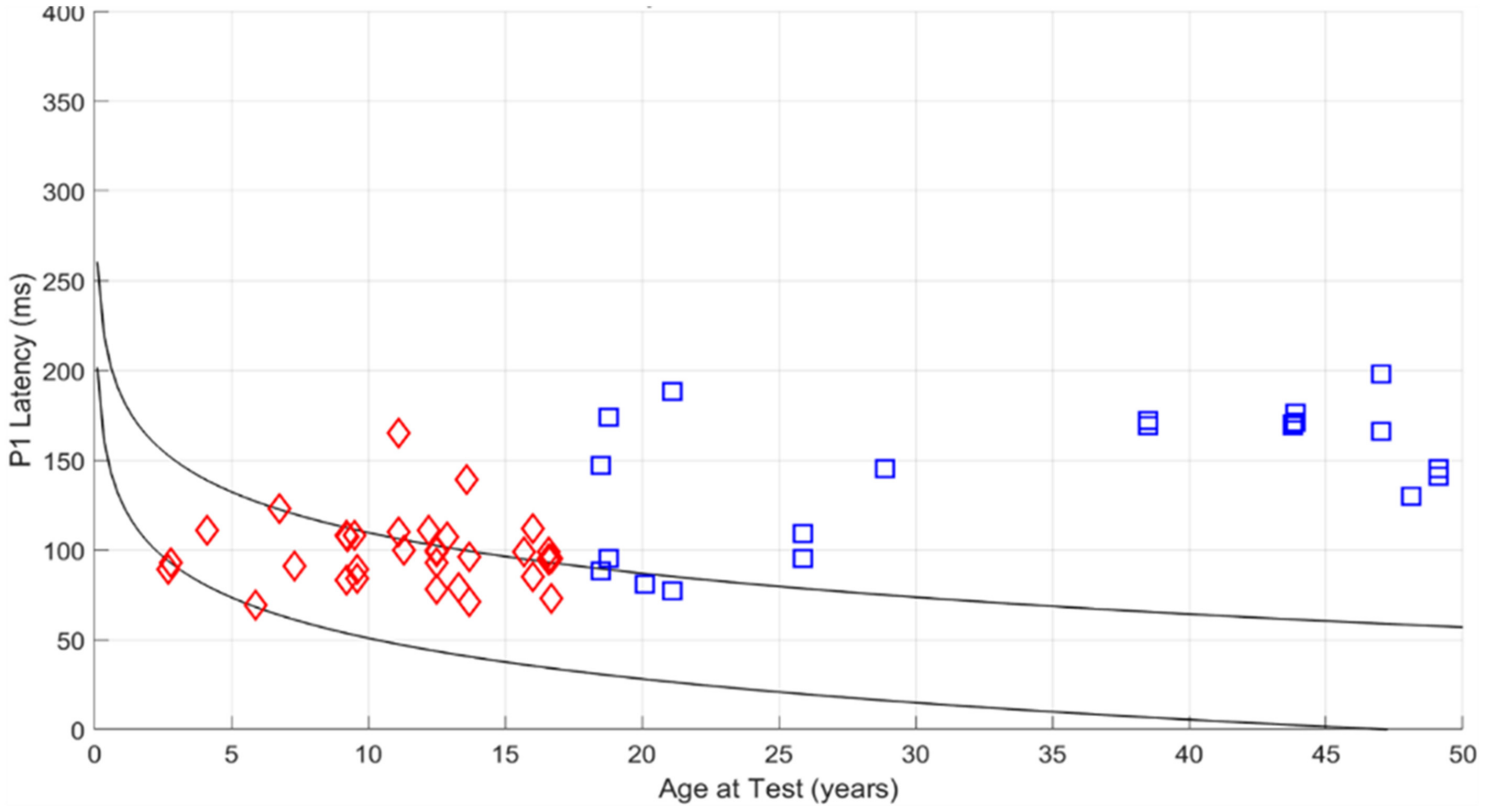
P1 latency as recorded directly from the CI as a function of current age in pediatric CI users (Red diamond mark) and in adults CI users (Blue square mark) shown in the P1 norms of subject with normal hearing and P1 recorded by scalp electrodes (Sharma et al., 2002). The two lines represents The line of best fit and the 95% confidence interval are superimposed on the raw data.

Figure 5 illustrates the peak latency values of the electrically evoked cortical auditory evoked potentials (eCAEPs) for all study participants, compared to age-matched normative data obtained from scalp electrode recordings in individuals with normal hearing (Sharma et al., 2002).

Among the 33 implanted ears of pediatric participants, 30 (91%) exhibited P1 latencies within the expected normative range for typically developing children. The remaining three children (9%) showed prolonged P1 latencies exceeding the upper normative threshold. Notably, two of these children were transitioned immediately into a signing environment, including communication via sign language, while the third was diagnosed with auditory neuropathy. All three demonstrated low performance levels on standardized auditory and language assessment scales.

In contrast, among the 21 implanted ears in the adult cohort, 16 (76%) presented with abnormally delayed P1 latencies relative to age norms. Only five adults (24%) exhibited P1 latencies within normal limits; all five had received cochlear implants at a very young age. These individuals demonstrated superior auditory and speech outcomes, as reflected in their CAP, SIR, and speech intelligibility scores (discussed in detail later). Conversely, adults with prolonged P1 latencies showed comparatively poor auditory and speech performance.

In this study, 33 ears from pediatric CI users were evaluated. Of these, 31 ears were implanted at an early age, ranging from 0.5 to 3.8 years, while the remaining two ears were implanted later, at 5.8 and 6.5 years of age. Within the adult cohort, seven ears received implants before the age of 4 years, one ear at 8.1 years, and 13 ears were implanted at ages above 14.8 years. Additionally, 11 ears were implanted during adulthood. Detailed demographic and implantation data are presented in Table 1.

**Figure 6:**
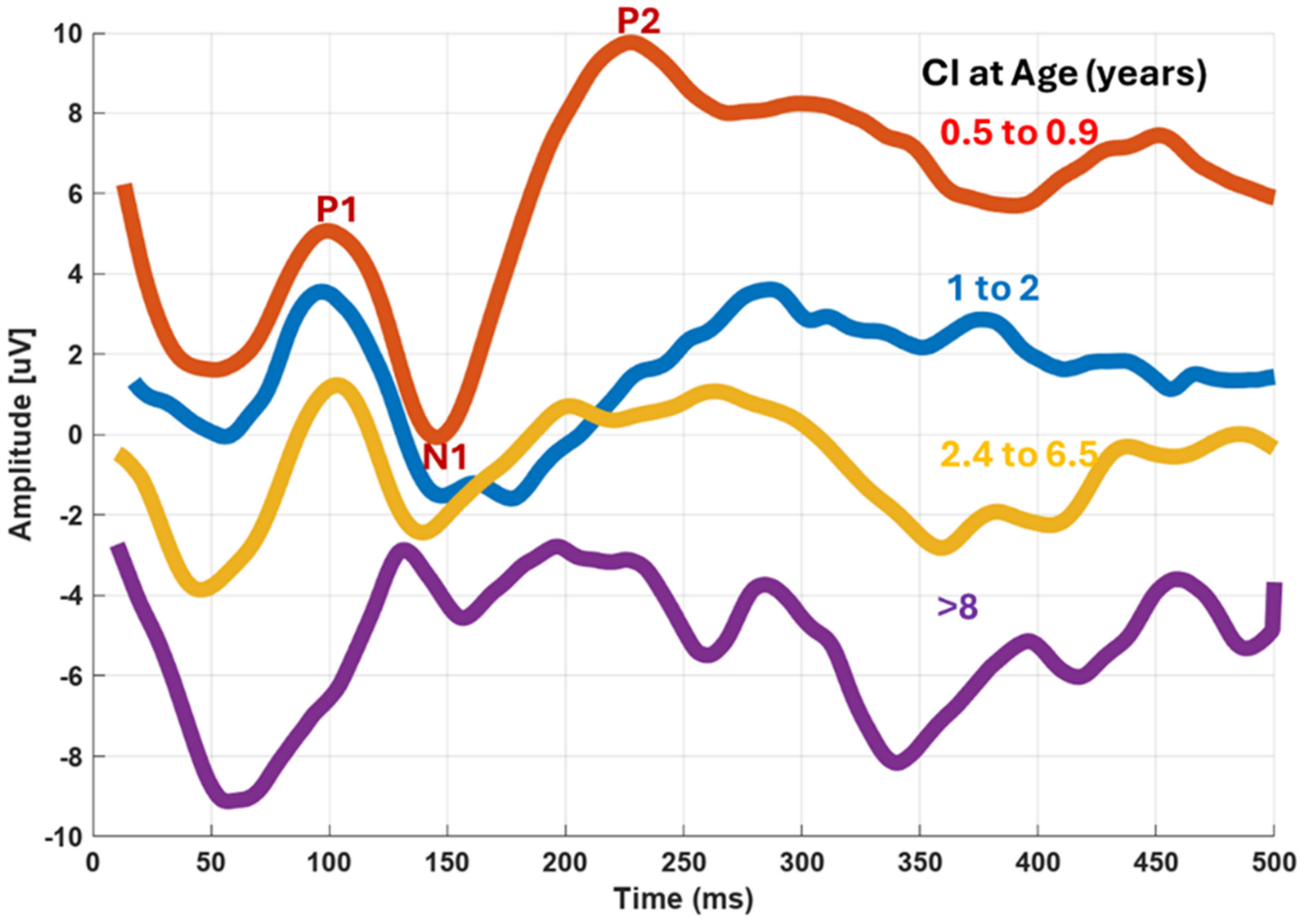
Grand average eCAEP waveforms for 54 CI ears grouped by age of implantation: 0.5–0.9 year, 1–2 years, 2.4–6.5 years, and beyond 8 years.

To capture clinically meaningful differences in age of implantation, participants were divided into four subgroups: < 1, 1–2, 2.4–6.5, and >8 years. The median P1 latencies were 99, 95, 98, and 169.5 ms, respectively. These intervals reflect key developmental milestones and typical clinical decision points for cochlear implantation. This stratification allows for a more precise evaluation of how early vs. late implantation impacts cortical response timing, beyond a simple early/late dichotomy. A Kruskal–Wallis test indicated a significant difference among groups (*H* = 30.09, *p* < 0.0001). *Post hoc* pairwise comparisons (Mann–Whitney *U* with Bonferroni correction) confirmed that the >8 years group had significantly longer latencies compared to all younger groups, while differences among the three younger groups were not statistically significant. These findings highlight the strong influence of early implantation on cortical auditory response timing. Figure 6 illustrates the grand average eCAEP responses of the study participants grouped by cochlear implantation timing, as follows: less than 1 year 11 implanted ears, between 1 and 2 years 16 ears, between 2.1 and 2.5 years, seven ears, from 2.9 to 6.5 years, eight ears and greater than 15 years 13 ears.

Table 3 summarizes the regression analysis examining the effects of current age (AgeNow) and age at cochlear implantation (Age_at_CI) on P1 and N1 latencies. The results indicate that Age_at_CI is a significant predictor for both components (*p* < 0.01), whereas AgeNow shows no significant effect (*p* > 0.39). This suggests that later implantation is associated with longer neural response latencies, while the participant's current age does not influence these measures.

**Table 3:** Regression coefficients and significance for AgeNow and age at CI as a predictors for P1-N1 latencies.

**Predictor**	**LP1_Coeff**	**LP1_p**	**LN1_Coeff**	**LN1_p**
Intercept	104.1542	0.0	157.845	0.0001
AgeNow	−0.3405	0.5978	−0.6995	0.3976
Age_at_CI	2.0574	0.0013	2.2673	0.0051

Age_at_CI was consistently significant; AgeNow showed no effect.

Similar statistical differences were observed when participants were categorized based on whether implantation occurred before or after language acquisition. Mann–Whitney *U*-test was used to compare latencies between participants implanted before language acquisition (Group 1) and after language acquisition (Group 2). For P1 latency: *U* = 28.50, *p* < 0.001. For N1 latency: *U* = 41.00, *p* < 0.001. Median latencies were substantially shorter in the pre-language group (P1 = 99 ms, N1 = 147.5 ms) compared to the post-language group (P1 = 169 ms, N1 = 211 ms). These results indicate a statistically significant difference, suggesting that implantation before language acquisition is associated with faster cortical auditory responses.

### Impact of implant use duration on cortical latencies

The duration of implant use was defined as the time elapsed between the date of cochlear implantation and the date of data collection, irrespective of the individual's daily usage pattern. Based on this definition, no significant effect of implant use duration was observed on the eCAEP components in either the pediatric group, the adult group, or the combined population. Spearman correlation analysis revealed a significant relationship between cochlear implant use duration (CI_Use) and cortical response latencies. Across the entire sample, longer CI use was associated with shorter latencies for both P1 (*r* = −0.375, *p* = 0.0067) and N1 (*r* = −0.429, *p* = 0.0017), indicating improved cortical processing with extended device experience.

When analyzed by age group, distinct patterns emerged. In children (< 18 years), correlations were weak and non-significant for P1 (*r* = −0.161, *p* = 0.395) and marginal for N1 (*r* = −0.357, *p* = 0.053), suggesting limited influence of CI use duration on latency within this group. Conversely, in adults (≥18 years), CI use showed strong and significant associations: P1 latency (*r* = −0.606, *p* = 0.0036) and N1 latency (*r* = −0.445, *p* = 0.043). These findings indicate that prolonged CI experience contributes substantially to faster cortical responses in adults, while in children, developmental factors may overshadow the effect of CI use.

### Associations between eCAEP and auditory and speech performances

In this study, CI user performance was assessed using two primary approaches. The first involved speech perception testing, including monosyllabic word recognition in the participants' native language under both quiet and noisy listening conditions. The second approach utilized clinician-rated scales: the Categories of Auditory Performance (CAP) and Speech Intelligibility Rating (SIR).

Speech perception testing was successfully conducted in 50 cochlear implant users. However, testing was not feasible in four pediatric cases due to clinical and developmental limitations: one child was too young for reliable assessment; another was both under the age threshold and diagnosed with global developmental delay; a third was diagnosed with auditory neuropathy spectrum disorder (ANSD); and the fourth was too young and also diagnosed with autism spectrum disorder (ASD).

**Figure 7:**
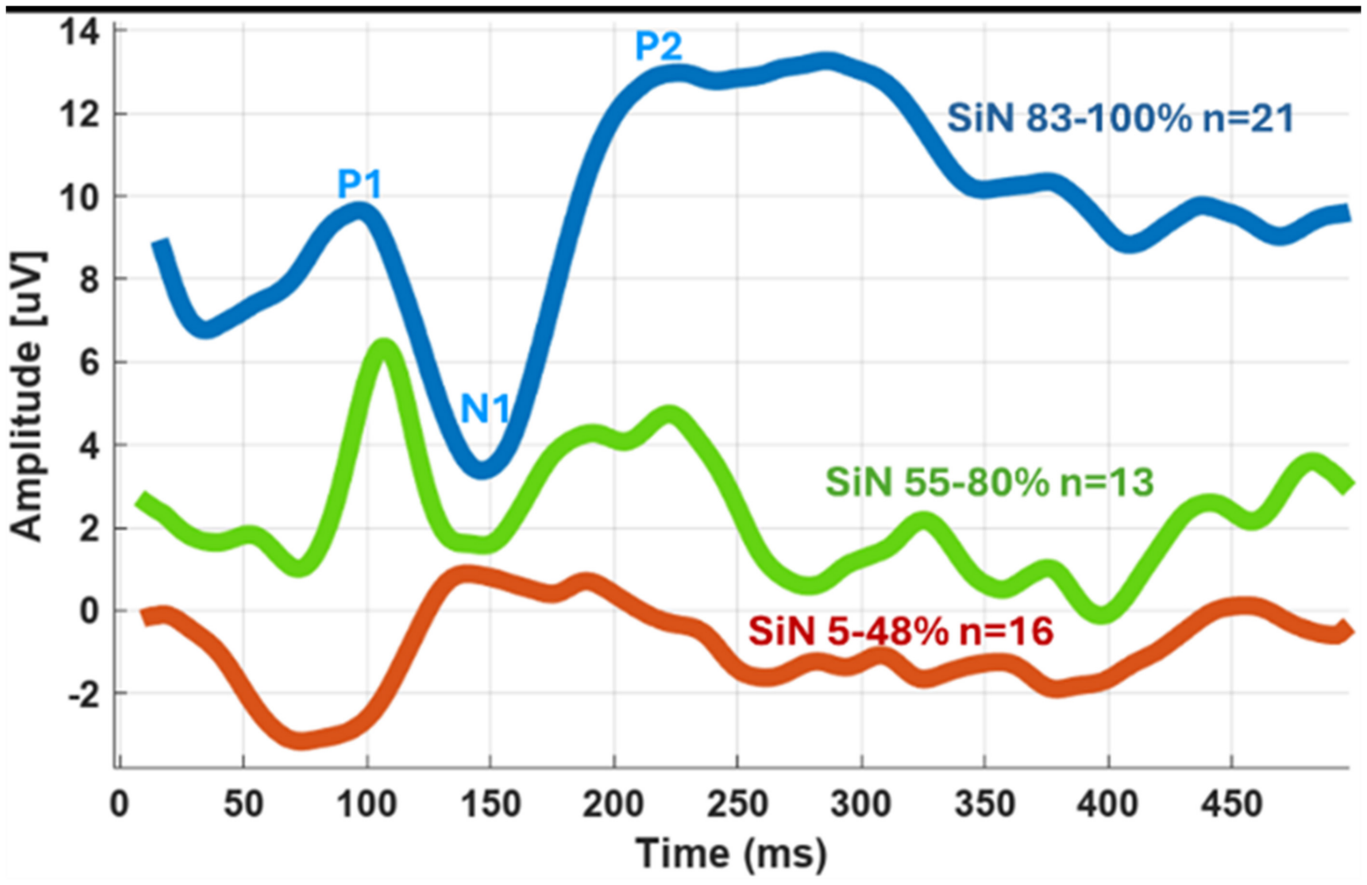
Grand average eCAEP waveforms for 51 CI ears grouped by Speech-in-Noise (SiN) scores: high (83%−100%), moderate (55%−88%), and low (5%−48%). Note the distinct eCAEP patterns across the SiN score groups.

Figure 7 presents the average eCAEP recordings for three groups of participants differing in their speech perception scores in noise (83%−100%; 55%−80%; 5%−48%). Implanted ears with good to very good speech perception in noise show an average eCAEP waveform with clear P1–N1 components, characterized by shorter latencies and higher amplitudes. In contrast, implanted ears with poorer speech perception demonstrate an average eCAEP waveform with longer latencies and reduced amplitudes.

**Figure 8:**
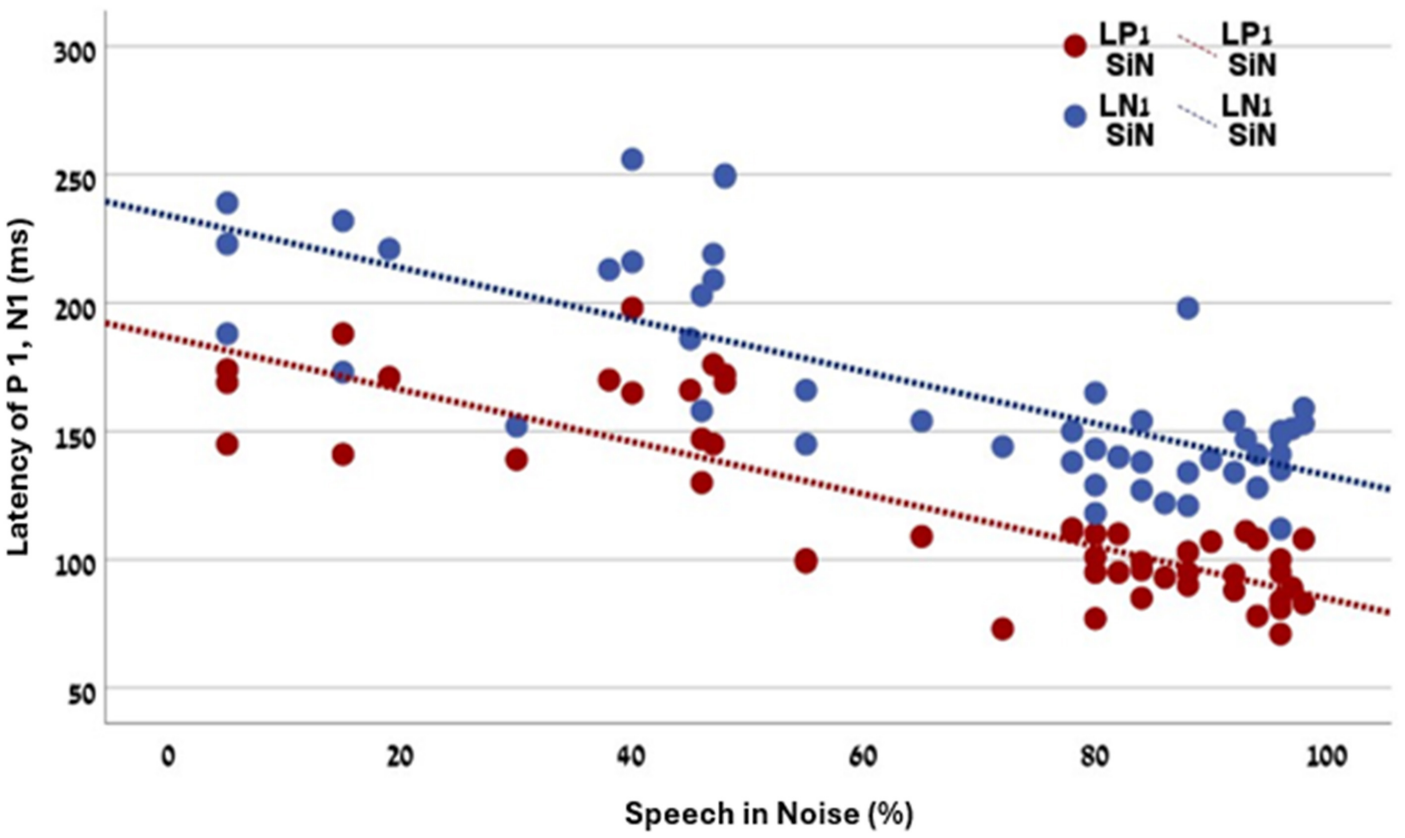
Combined scatter plot for the relationship between Speech in noise and P1 and N1 latencies. Both P1 and Ni latencies strongly correlated with the speech intelligibility in noise (*r* = −0.751 and −0.633, respectively).

A Mann–Whitney *U*-test compared speech perception between children (< 18 years) and adults (≥18 years). Results showed significant differences in both conditions. For speech in noise (SiN), children achieved markedly higher scores (median = 88.0) than adults (median = 46.0; *U* = 500.50, *p* < 0.00001). For speech in quiet (SiQ), children also outperformed adults (median = 92.0 vs. 75.0; *U* = 446.00, *p* = 0.00066). These findings highlight the advantage of early implantation, particularly in noisy environments. Figure 8 illustrates the relationship between auditory cortical latencies (P1 and N1) and SiN performance. Longer latencies were associated with poorer SiN scores, indicating that delayed cortical processing negatively impacts speech understanding in challenging conditions. Spearman correlations confirmed these trends across all eCAEP measures. P1 latency (LP1) showed strong negative correlations with SiN (*r* = −0.751, *p* < 0.0001) and SiQ (*r* = −0.632, *p* < 0.0001). N1 latency (LN1) also correlated negatively with SiN (*r* = −0.633) and SiQ (*r* = −0.547). P1 and N1 amplitude measures were positively associated with performance in speech in noise (*r* = 0.34 and 0.53 respectively). These results confirm that latency and amplitude of cortical responses are key predictors of speech perception, with latency effects most pronounced for SiN. Further analysis revealed that age at cochlear implantation strongly influenced outcomes. Later implantation correlated with poorer SiN (*r* = −0.747, *p* < 10^−9^) and SiQ (*r* = −0.632, *p* < 10^−6^), underscoring the critical role of early implantation.

Figure 8 illustrates the relationship between auditory cortical latencies (P1 and N1) and SiN performance. Longer latencies were associated with poorer SiN scores, indicating that delayed cortical processing negatively impacts speech understanding in challenging conditions. Spearman correlations confirmed these trends across all eCAEP measures. P1 latency (LP1) showed strong negative correlations with SiN (*r* = −0.751, *p* < 0.0001) and SiQ (*r* = −0.632, *p* < 0.0001). N1 latency (LN1) also correlated negatively with SiN (*r* = −0.633) and SiQ (*r* = −0.547). P1 and N1 amplitude measures were positively associated with performance in speech in noise (*r* = 0.34 and 0.53 respectively). These results confirm that latency and amplitude of cortical responses are key predictors of speech perception, with latency effects most pronounced for SiN. Further analysis revealed that age at cochlear implantation strongly influenced outcomes. Later implantation correlated with poorer SiN (*r* = −0.747, *p* < 10^−9^) and SiQ (*r* = −0.632, p < 10^−6^), underscoring the critical role of early implantation.

In order to identify the effects of age at CI and current age on SiN, we conducted a regression analysis to evaluate the relative contribution of each parameter to speech-in-noise (SiN) scores. Both variables were entered into the same model. The results showed that age at implantation had a stronger effect on SiN performance (β = −0.94, *p* = 0.081) compared to current age (β = −0.62, *p* = 0.288), although neither reached conventional significance when combined in the same model. The model explained approximately 60% of the variance in SiN scores (*R*^2^ = 0.605). These findings, together with the strong negative correlations observed between SiN and age at implantation (*r* = −0.77, *p* < 0.0001) and between SiN and current age (*r* = −0.76, *p* < 0.0001), confirm that later implantation and older age are associated with poorer speech-in-noise performance. Importantly, age at implantation remains the more influential predictor, reinforcing the critical role of early implantation in optimizing auditory outcomes.

**Figure 9:**
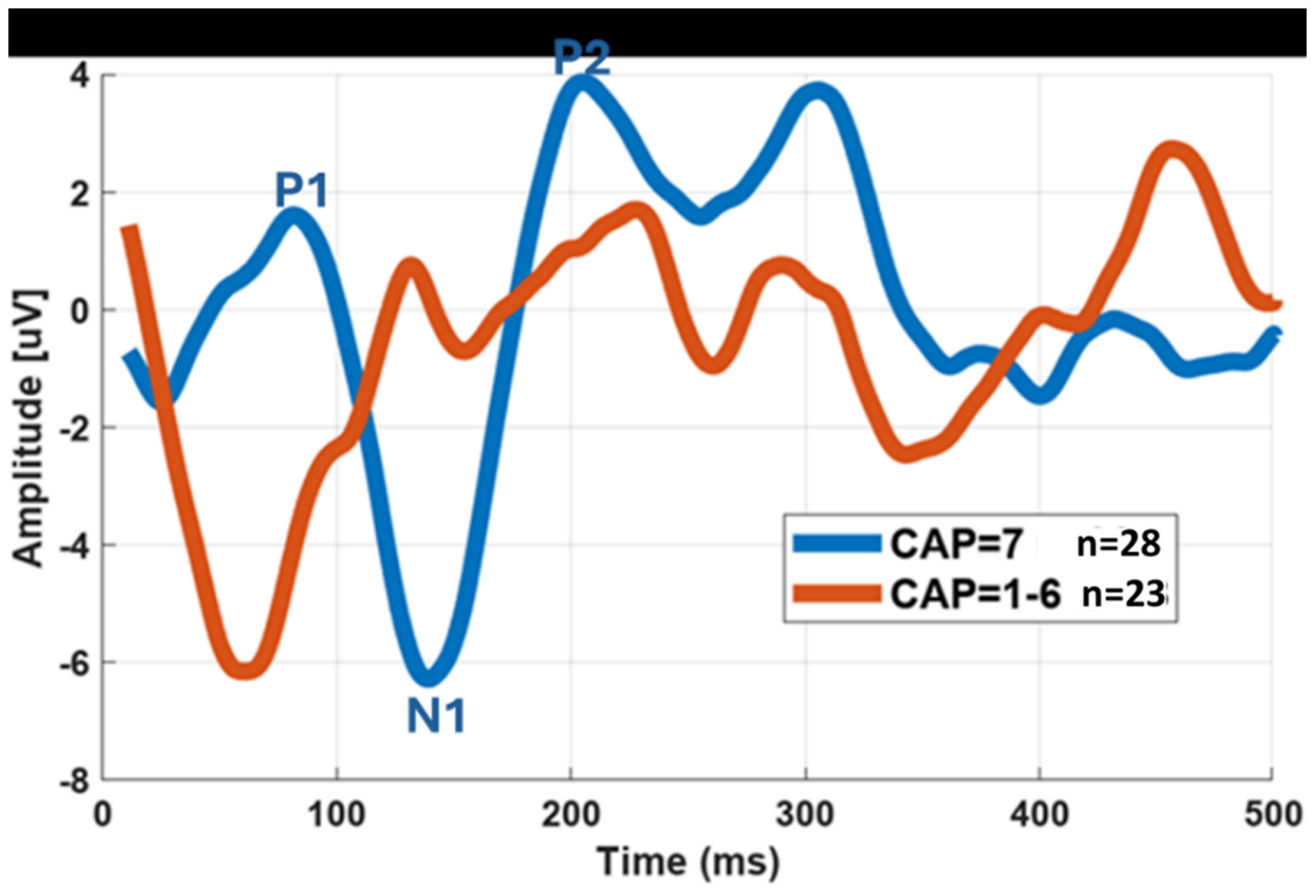
Grand average eCAEP recordings of CI users with CAP scores within the normative range (score of 7) compared to those with lower CAP scores (less than scale 7).

In addition to speech intelligibility, analysis was also conducted to the CAP and SIR scores of the CI participants. Because sample sizes varied, scores were grouped into two categories for statistical analysis: normative performance (CAP = 7; SIR = 5) and sub normative performance (scores below these thresholds). In the pediatric group, CAP scores were distributed as follows: 21 children scored 7, one scored 6, one scored 5, three scored 4, one scored 3, and one scored 1. For SIR, 26 children achieved the maximum score of 5, one scored 4, three scored 3, and one scored 1. CAP or SIR evaluations were not feasible for two children due to age (under 3 years) and, in one case, a diagnosis of autism spectrum disorder (ASD). In the adult group, CAP scores were: 7 (*n* = 4), 6 (*n* = 6), 5 (*n* = 5), 4 (*n* = 2), 3 (*n* = 1), and 1 (*n* = 3). SIR scores were: 5 (*n* = 6), 4 (*n* = 7), 3 (*n* = 5), and 1 (*n* = 3). Figure 9 illustrates the average eCAEP recordings of all participants with CAP scores within the normative range (score of 7) compared to those with lower CAP scores. While the general eCAEP waveform pattern was similar between the two groups, participants with poorer auditory performance exhibited longer P1–N1 latencies and lower amplitudes than those in the normative performance group.

Tables 4, 5 summarize the Mann–Whitney *U*-test results comparing cortical measures and implantation age across CAP and SIR functional categories. Significant differences were observed for all variables, indicating that better functional outcomes are associated with earlier implantation, shorter latencies, and larger amplitudes.

**Table 4:** CAP category comparison (normal = score = 7; *n* = 28 vs. abnormal score ≤ 6; *n* = 24).

**Variable**	**Median normal (CAP=7)**	**Median abnormal (CAP < = 6)**	**U**	***p*-value**
Age_at_CI	1.50	27.8	58.0	2.88 × 10^−6^
LP1	95.0	147.0	33.0	2.01 × 10^−7^
LN1	141.0	203.0	71.5	1.10 × 10^−5^
PPP1	7.11	2.80	416.5	0.0059
PPN1	9.70	3.10	505.0	4.36 × 10^−6^

**Table 5:** SIR category comparison (normal score = 5; *n* = 31 vs. abnormal score < 5; *n* = 20).

**Variable**	**Median normal (SIR = 5)**	**Median abnormal (SIR < 5)**	***U***	***p*-value**
Age_at_CI	1.55	32.0	35.0	5.82 × 10^−7^
LP1	95.0	167.5	20.0	1.06 × 10^−7^
LN1	140.5	211.0	25.5	2.01 × 10^−7^
PPP1	6.05	2.95	378.5	0.0214
PPN1	8.75	2.90	487.5	3.81 × 10^−6^

**Figure 10:**
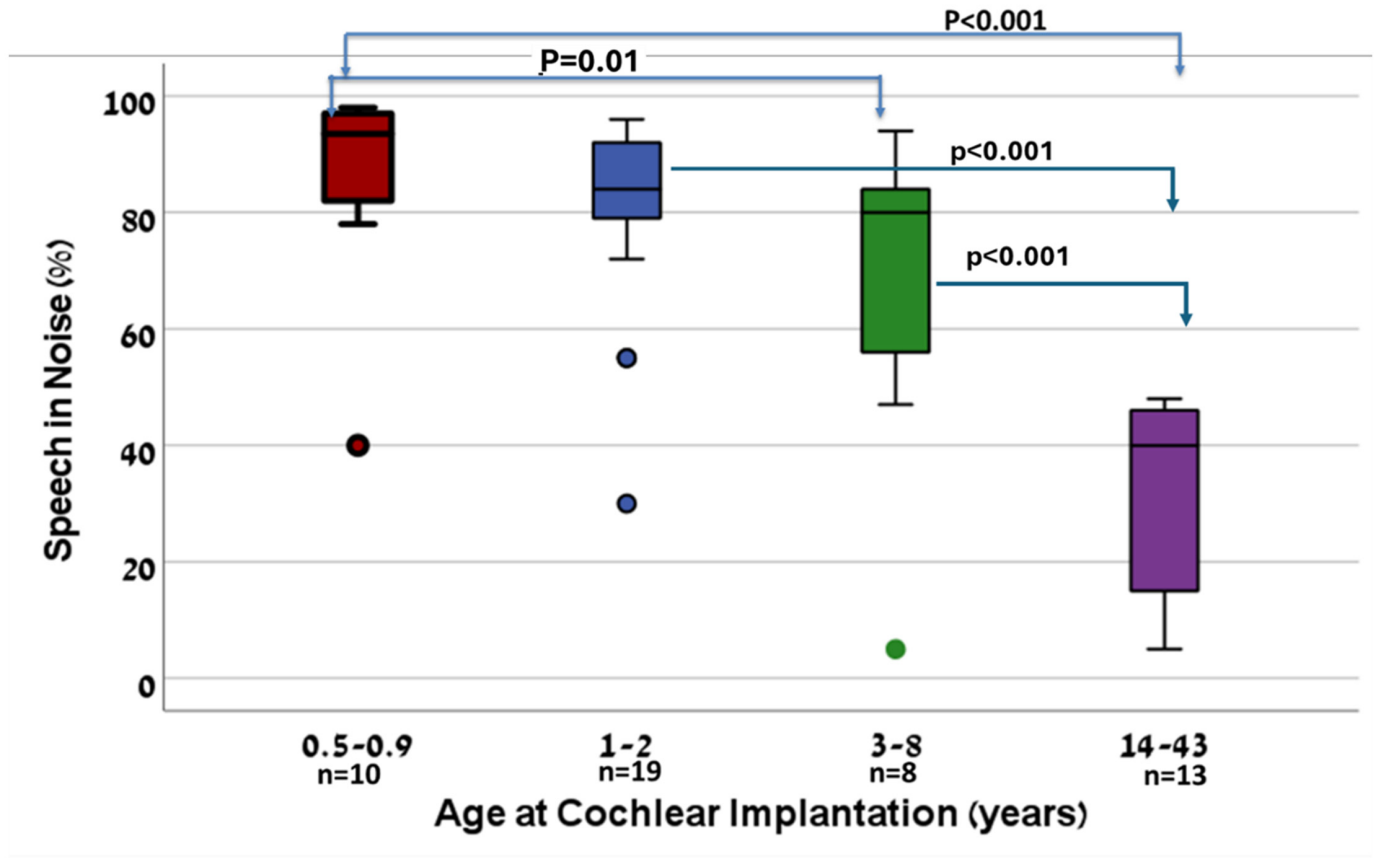
Speech-in-noise performance by age at cochlear implantation. Box plots represent four age groups at implantation: 0.5–0.9, 1–2, 3–8, and 14–43 years.

Figure 10 illustrates the relationship between age at cochlear implantation and speech-in-noise performance. Participants were divided into four age groups: implantation before 1 year of age, between 1 and 2 years, between 3 and 8 years, and between 14 and 43 years. The stratification intervals for age at implantation in the speech performance analysis were selected to ensure clinically meaningful groupings and adequate sample sizes, which differ from the finer divisions used for latency analysis that reflect neurodevelopmental milestones. This rationale has been clarified to avoid confusion. A clear trend is observed, indicating that earlier implantation is associated with better speech-in-noise outcomes. Specifically, the median and interquartile ranges of speech-in-noise scores decrease progressively with later implantation age. This trend reached statistical significance only when comparing the earliest implantation group ( ≤ 1 year) with the group implanted between 3 and 8 years (*p* < 0.04) and was highly significant when compared to the latest implantation group (14–43 years; *p* < 0.001). The median score of the late-implanted group was substantially lower than that of all early-implanted groups. Notably, outliers were identified in the early implantation groups ( ≤ 1 and 1–2 years). Two cases involved children who were transitioned to sign language communication shortly after implantation and demonstrated limited device use. Another outlier, despite achieving maximum CAP and SIR scores, showed unexpectedly poor speech-in-noise performance (55%) with no clear explanation. An additional outlier in the 3–8 years group also showed a deviant score, though the underlying cause remains unclear.

Participants were grouped according to age at cochlear implantation: < 1, 1–2, 3–8, and 14–43 years. Median SiN scores for these groups were 93%, 90%, 80%, and 39%, respectively. A Kruskal–Wallis test revealed a significant overall difference among groups (*H* = 29.18, *p* < 0.0001). *Post hoc* pairwise comparisons using Mann–Whitney *U*-tests with Bonferroni correction indicated that the late-implanted group (14–43 years) performed significantly worse than all three early-implanted groups (*p* ≤ 0.0001), whereas differences among the early-implanted groups (< 1 year, 1–2 years, and 3–8 years) was also statistically significant (*p* =0.01). A clear trend is observed, showing that earlier implantation is associated with better speech-in-noise outcomes. These findings confirm that earlier implantation is strongly associated with better speech-in-noise perception.

**Figure 11:**
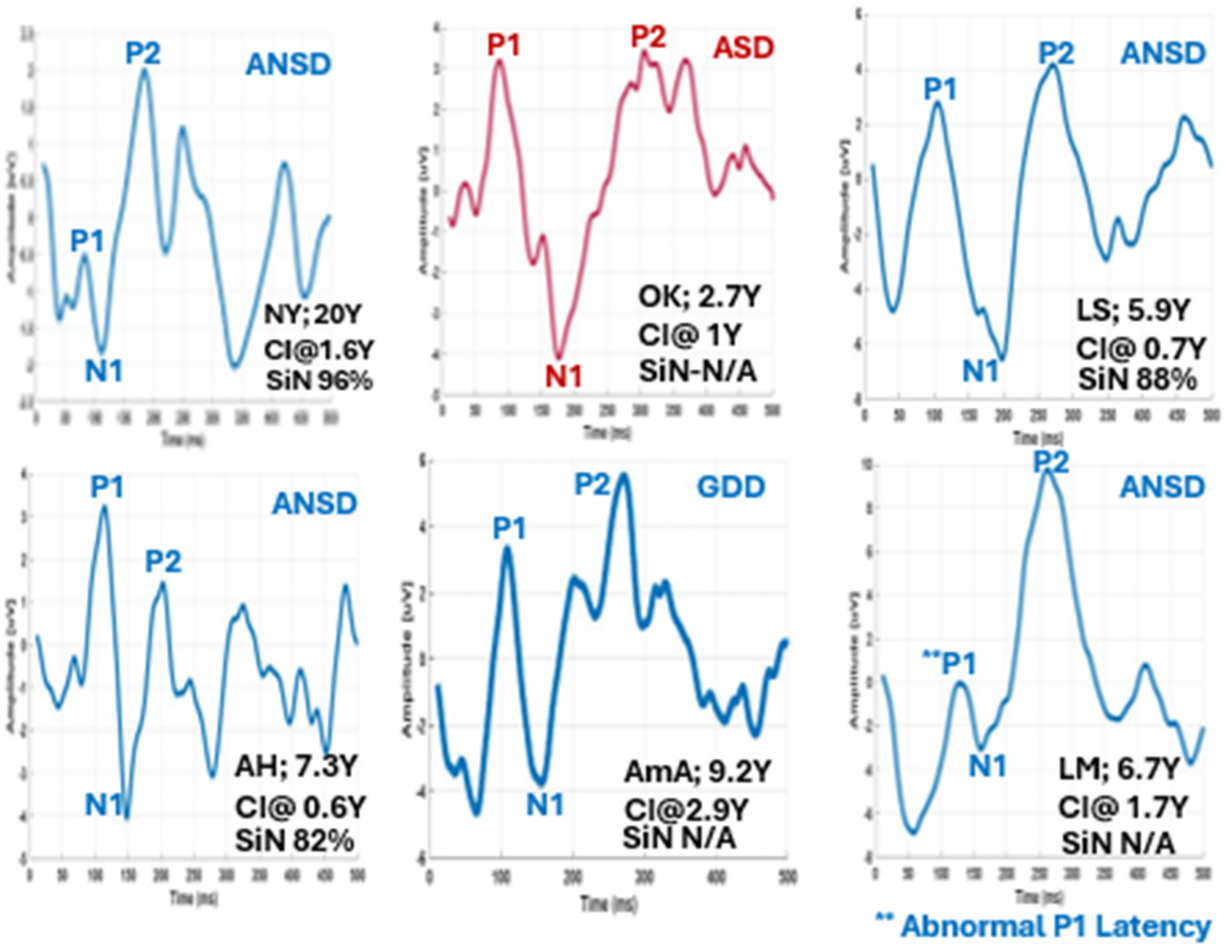
eCAEP waveforms from six children with various etiologies, all of whom were implanted early in life. Recordings include four children with Auditory Neuropathy Spectrum Disorder (ANSD), one of whom showed delayed P1 latency and poor auditory outcomes, while the others exhibited age-appropriate P1 latencies and good speech perception in noise. Two additional children with Global Developmental Delay (GDD) and autism spectrum disorder (ASD) demonstrated normal-range P1 latencies, but their speech and auditory performance could not be assessed due to their neurological conditions.

Figure 11 shows the eCAEP recordings of six participants with underlying conditions that could affect cortical response patterns and cochlear implant outcomes. All were implanted early in life. Among the four participants diagnosed with ANSD, one child demonstrated a prolonged P1 latency beyond the normal range for her age, along with low SIR and CAP scores. Speech perception in both quiet and noisy environments could not be assessed in her case. In contrast, the other three children with ANSD showed age-appropriate P1 latencies and good speech perception in noise. The remaining two children, diagnosed with Global Developmental Delay (GDD) and ASD, displayed eCAEP waveforms similar to those typically seen in normally developing children, including age-appropriate P1 latencies. However, their speech and auditory performance could not be evaluated due to the nature of their neurological conditions.

**Figure 12:**
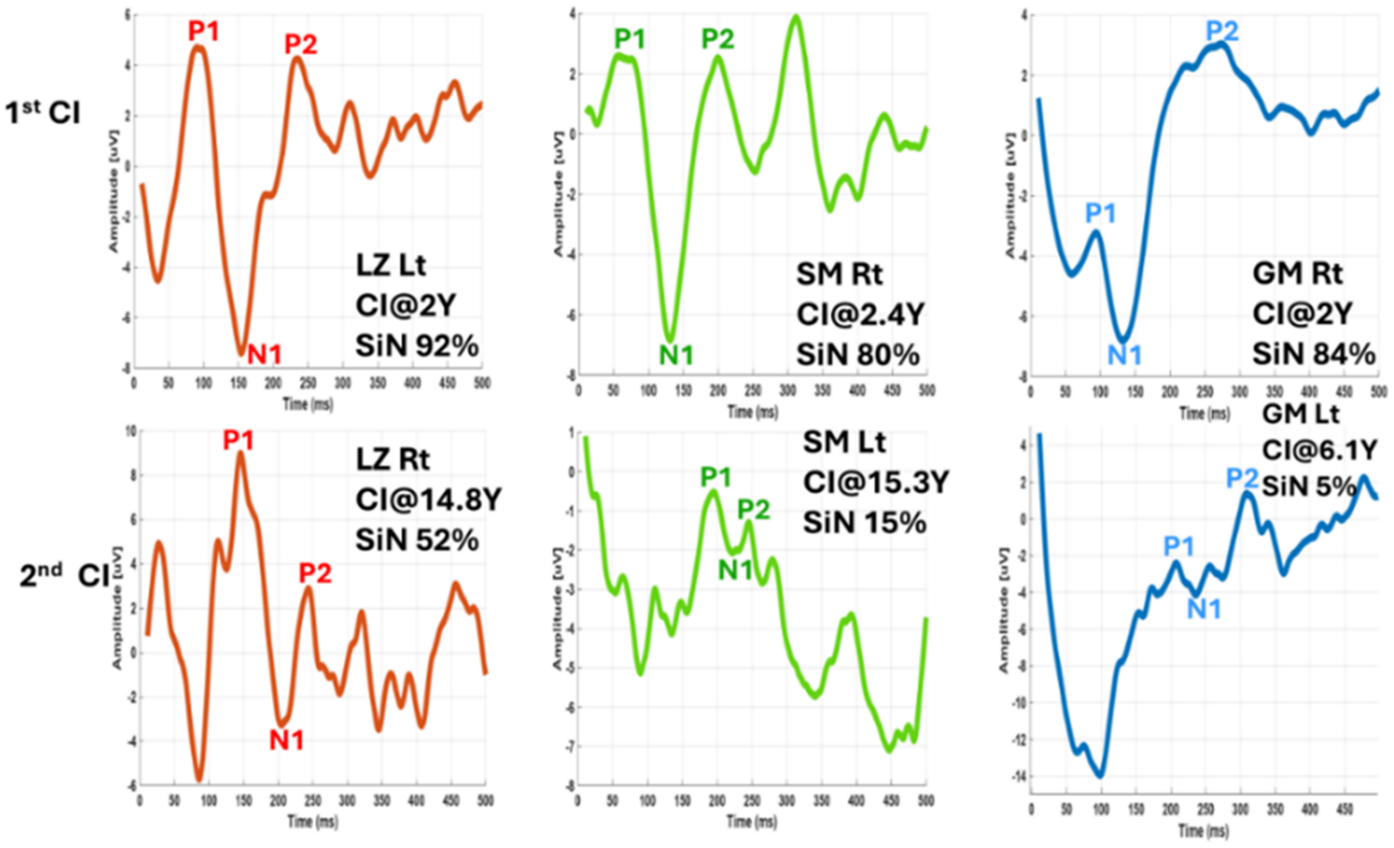
Intracochlear eCAEP waveforms from a sequentially implanted bilateral CI user. The first-implanted ear shows earlier latencies and better speech performance, highlighting the impact of earlier auditory stimulation.

To demonstrate the impact of sequential implantation on speech understanding in noise, Figure 12 highlights three children, the only ones in our sample who received bilateral cochlear implants before acquiring language. In each case, however, there was a gap of several years between implantation of the first ear and the contralateral ear. As shown, the ears implanted earlier in life consistently performed better in speech-in-noise tasks than the later-implanted ears, where the delay ranged from 4.1 to nearly 13 years after the first implantation.

Among all adult participants, eight individuals underwent sequential bilateral implantation. Their current ages ranged from 18.5 to 49.1 years, and the interval between implants varied from 3.7 to 18.7 years (mean ± SD: 9.7 ± 4.7 years). A significant negative correlation was found between speech perception performance and P1 latency recorded from the hemisphere contralateral to the tested ear, with *r* = −0.58 for speech in quiet and *r* = −0.70 for speech in noise. However, when correlating the same contralateral P1 latency values with speech perception performance of the *ipsilateral* ear (i.e., the ear on the same side as the recorded hemisphere), no significant correlations were observed. These results suggest that, despite the large time gaps between implantations, cortical dominance remained contralateral to the ear stimulated by the implant, independent of the timing of implantation.

To determine whether eCAEP latency provides predictive value for speech in noise beyond age at implantation, we conducted supplementary regression analyses. Age at implantation alone explained 59.5% of the variance in speech-in-noise performance (*R*^2^ = 0.595). When LP1 latency was added to the model, the explained variance increased to 72.8% (Δ*R*^2^ = +0.133), indicating a substantial improvement in prediction. LP1 also demonstrated a stronger correlation with SiN (*r* = −0.835) compared to age at implantation (*r* = −0.772). These findings confirm that eCAEP provides clinically relevant information beyond age at implantation.

## Discussion

Building on previous work demonstrating the feasibility of recording electrically evoked cortical auditory evoked potentials (eCAEPs) directly through the cochlear implant (CI) system in response to brief electrical stimuli (HabibAllah et al., 2025), the present study investigated how age at implantation, etiology of hearing loss, and comorbid conditions influence the morphology and latency of eCAEPs. It further explored how these neural markers relate to behavioral measures of speech perception and auditory function. The study included a diverse cohort of 33 pediatric and 21 adult CI users, enabling comparative analysis across developmental stages and clinical backgrounds.

A particularly notable finding from the intracochlear recording, especially among adult participants, was the presence of a split P2 component, characterized by two consecutive P2 peaks. This dual morphology resembles scalp-recorded responses in normal-hearing individuals exposed to voice pitch changes (Davis et al., 1966; Steinmetzger and Rupp, 2024) and supports the hypothesis that P2 comprises distinct subcomponents: an early P2a likely originating in the lateral auditory cortex, and a later P2b involving broader frontotemporal auditory-association areas. These subcomponents may reflect hierarchical stages of auditory processing, from initial sensory encoding to higher-order evaluation of sound features.

Similar P2 splitting has been previously associated with increased auditory processing effort in hearing aid users with asymmetric input (Ross and Tremblay, 2009; Bertoli et al., 2011), suggesting that the phenomenon observed in CI users may reflect neural compensation mechanisms for degraded or incomplete auditory input. Interestingly, such dual-peak P2 morphology has not been reported in scalp-recorded eCAEPs elicited by electrical stimulation in CI users (Liebscher et al., 2018; Távora-Vieira et al., 2021; Callejón-Leblic et al., 2022). This discrepancy may be attributed to the superior spatial resolution provided by the intracochlear montage, which enables more precise detection of subtle cortical dynamics. The observed patterns point to the potential of intracochlear recordings as a tool for exploring auditory cortical mechanisms, a notion that warrants further investigation.

Analysis of eCAEP responses revealed marked differences between pediatric and adult CI users. Children who received implants at an early age exhibited significantly shorter latencies and greater amplitudes in the P1, N1, and P2 components compared to those implanted later. Most P1 latencies in this group fell within age-appropriate developmental ranges. In contrast, nearly all adult participants, in which the vast majority were suffered from demonstrated prolonged P1 latencies, frequently exceeding normative thresholds, indicative of delayed or incomplete cortical maturation. These findings are consistent with previous scalp-recorded CAEP studies (Sharma et al., 2002, 2005), reinforcing the importance of early auditory input in shaping cortical responsiveness. They align with the concept of a critical period for auditory neuroplasticity (Kral and Tillein, 2006; Glennon et al., 2020).

Notably, our data suggest that the concept of “hearing age”-defined as the cumulative duration of auditory experience since activation of the implant-holds clinical relevance only when implantation occurs within the sensitive period and is accompanied by consistent auditory stimulation and exposure to spoken language. The significance of sustained auditory and linguistic input is further underscored by cases in which children, despite early implantation, transitioned to sign-language-based communication or demonstrated inconsistent device use. These individuals failed to exhibit expected auditory cortical development. Importantly, this suggests that the critical period for neuroplasticity-while offering a window of heightened sensitivity to auditory input-can also regress if the auditory system is deprived of consistent stimulation or redirected toward alternative modes of communication. In such cases, the shift to visual-manual language modalities may interrupt or even reverse auditory cortical maturation, leading to stagnation in auditory development despite early access to cochlear implantation. These observations emphasize that early implantation alone is insufficient; its benefits are contingent upon continuous and enriched auditory experience within a spoken language environment.

Regression analyses revealed distinct patterns in the relationship between age at implantation and cortical response latencies. For the P1 component, age at implantation accounted for approximately 56% of the variance in latency (*R*^2^ = 0.558, *p* < 0.001). This robust association highlights the critical role of early implantation in promoting timely cortical auditory processing. Similarly, the regression model for the N1 component yielded a statistically significant but slightly weaker effect, with age at implantation explaining about 40% of the variance (*R*^2^ = 0.4, *p* = 0.002).

The strong correlation between age at implantation and cortical response timing underscores the fundamental principle of auditory neuroplasticity. Shorter P1 latencies in early-implanted children reflect more efficient activation of primary auditory cortex during the critical period, when synaptic connectivity and cortical circuits remain highly adaptable. In contrast, prolonged latencies observed in late-implanted individuals suggest delayed cortical synchronization and reduced temporal precision, likely due to long-standing auditory deprivation and cross-modal reorganization. These changes indicate that once the sensitive window closes, auditory pathways become less responsive to electrical stimulation, limiting the potential for full cortical recovery.

The stability of P1 latency across the youngest implantation groups (< 1 year, 1–2 years, and up to 6.5 years) suggests that early auditory input supports near-normal cortical maturation, whereas the sharp increase beyond 8 years reflects a neurodevelopmental threshold beyond which compensatory mechanisms cannot fully restore typical timing. This pattern highlights that cortical auditory processing is not only experienced-dependent but also time-sensitive, reinforcing the concept that early implantation is essential for establishing robust neural representations of sound.

From a clinical perspective, these findings emphasize that intervention strategies should prioritize implantation within the first year of life to leverage maximal cortical plasticity. Beyond this period, rehabilitation must account for altered cortical dynamics, focusing on strategies that enhance attentional control and multisensory integration to compensate for reduced auditory efficiency.

Overall, these findings underscore the enduring influence of early sensory experience on cortical architecture-a principle well-established in the literature on neuroplasticity and sensory development (Jeong et al., 2018; Purcell et al., 2021; Jafari et al., 2024).

The prolonged latencies and reduced amplitudes observed in late-implanted adults are consistent with cortical reorganization driven by cross-modal plasticity, wherein auditory cortical regions are recruited by non-auditory modalities (Sharma et al., 2009; Lee et al., 2001). Even with extended device use, the persistence of these deficits suggests that neuroplastic recovery may be limited once critical developmental windows have closed.

Further evidence for the importance of early and consistent auditory input emerged from cases of sequential bilateral implantation. In these individuals, the first implanted ear consistently demonstrated shorter eCAEP latencies and superior functional outcomes, regardless of the delay between implantations. These ear-specific differences likely reflect patterns of hemispheric dominance, with the auditory cortex contralateral to the first implanted ear undergoing more robust maturation-a phenomenon previously documented (Shader et al., 2022; Kraaijenga et al., 2017). These findings reinforce the notion that cortical auditory development is not only time-sensitive but also input-specific, with early stimulation playing a pivotal role in shaping hemispheric specialization.

Our analyses consistently demonstrated that current age was not a significant predictor of cortical response latencies once at implantation was considered, highlighting the dominance of early auditory experience over chronological maturation. Neural plasticity in the auditory system is highly time-sensitive: the critical period for establishing efficient cortical pathways occurs early in life and depends on timely sensory input. After this window closes, cortical architecture stabilizes, and additional years of age do not substantially alter latency patterns. Thus, auditory development is experience-driven rather than age-driven, reinforcing the clinical priority of early implantation. Age at implantation strongly predicted cortical latencies because these measures reflect fundamental neurodevelopmental processes during sensitive periods. In contrast, speech-in-noise performance is shaped by multiple higher-order factors-such as language experience, cognition, and listening strategies-reducing the relative impact of implantation age. This explains why age at implantation a robust predictor for latency measures was but only a modest predictor for functional speech outcomes.

However, although age at implantation is a strong predictor of auditory and speech outcomes, it does not provide direct evidence of cortical auditory processing. eCAEP recordings offer objective insight into neural responsiveness and maturation, enabling clinicians to monitor auditory development and identify variability among individuals beyond demographic factors. It should be noted that the primary conclusions of this study apply to prelingually deafened participants, as their outcomes are most strongly influenced by early auditory experience. *Participants with* congenital or prelingual hearing loss who were implanted postlingually were included in the overall analysis. However, given their unique auditory histories and residual language experience, these findings should be interpreted as exploratory and approached with caution.

Interestingly, our results diverge from animal studies that report ipsilateral dominance following prolonged unilateral stimulation (Kral et al., 2013). In contrast, contralateral dominance was preserved in our human cohort, even among adults with delayed and asymmetric implantation histories. This discrepancy may reflect fundamental interspecies differences. Unlike animals raised in complete sensory deprivation, many of our participants retained residual hearing and used hearing aids in the non-implanted ear, thereby maintaining bilateral auditory input and mitigating extreme cortical asymmetry. These findings suggest that although cross-modal plasticity occurs, the adult human brain retains an inherent organizational bias toward contralateral auditory processing.

This observation highlights the importance of considering naturalistic auditory experiences and pre-implant hearing history when extrapolating data from animal models to clinical populations.

Of particular clinical and neurobiological relevance are cases involving Auditory Neuropathy Spectrum Disorder (ANSD), Autism Spectrum Disorder (ASD), and Global Developmental Delay (GDD). Among the three children and one adult diagnosed with ANSD, all but one exhibited age-appropriate eCAEP patterns, supporting previous evidence that cochlear implantation in postsynaptic ANSD (synaptopathy) can facilitate typical auditory cortical development (Attias et al., 2017; Nassar et al., 2020; Sahwan et al., 2024). However, one child with ANSD showed delayed P1 latency and poor auditory progress despite early bilateral implantation, underscoring the role of inter-individual variability and the potential contribution of central auditory processing deficits beyond peripheral pathology.

Similarly, children diagnosed with ASD and GDD demonstrated normal eCAEP responses yet exhibited poor speech and auditory outcomes. This dissociation suggests that intact activation of the primary auditory cortex-as indicated by typical P1 latency-does not necessarily translate into functional success. These findings align with recent research indicating that deficits in cognition, attention, and multisensory integration can significantly impair outcomes, even in the presence of adequate auditory input (Morlet et al., 2023). Therefore, while eCAEPs may serve as useful biomarkers of cortical auditory plasticity, their interpretation must be contextualized within the broader framework of cognitive and neurodevelopmental status.

The observed association between longer implant use and shorter cortical latencies, particularly in adults, suggests experience-dependent plasticity within the auditory system. While early implantation remains the dominant factor for achieving near-normal cortical timing, extended device use appears to facilitate gradual improvements in neural synchrony even beyond the critical period. This is likely to reflect adaptive changes in cortical networks as repeated electrical stimulation strengthens synaptic efficiency and refines temporal coding. In adults, the strong correlations between implant use duration and both P1 and N1 latencies indicate that prolonged auditory experience can partially counteract the effects of long-term deprivation. These improvements may involve reactivation of dormant auditory pathways and enhanced integration within higher-order cortical areas responsible for speech processing. Conversely, the weak and non-significant correlations in children suggest that developmental factors overshadow the contribution of device experience during early years, as cortical maturation is primarily driven by age at implantation and linguistic exposure rather than cumulative stimulation time.

Clinically, these findings highlight the importance of consistent device use and long-term auditory engagement for late-implanted individuals. While early implantation offers the greatest neuroplastic advantage, extended CI experience can still promote measurable cortical gains, supporting the role of sustained rehabilitation and auditory training in optimizing outcomes for adult users.

Previous research has reported mixed findings regarding the influence of cochlear implant (CI) use duration on cortical auditory evoked potentials. Several studies observed significant improvements in latency with prolonged CI experience, particularly in adults, supporting experience-dependent plasticity beyond the critical period (Bogdanov et al., 2024; Lee et al., 2020; Deniz, 2025; Ghiselli et al., 2016; Bernhard et al., 2021). These improvements are thought to reflect gradual strengthening of cortical networks and enhanced temporal coding through sustained electrical stimulation. Conversely, other studies found no or minimal correlation between CI use duration and cortical latency, emphasizing that age at implantation and duration of auditory deprivation remain the dominant predictors of cortical timing (Schwab, 2015; Silva et al., 2013; Távora-Vieira et al., 2022). This variability suggests that while extended device use can promote cortical adaptation in late-implanted individuals, its effect is secondary to early auditory input and consistent linguistic exposure.

The second objective of this study was to examine how the electrophysiological properties of intracochlear eCAEPs-recorded directly via the cochlear implant system-relate to auditory and language performance in the implanted ear. Specifically, we evaluated the influence of current age, age at implantation, and duration of device use on speech and auditory outcomes, using two complementary approaches. The first involved global functional measures, namely the CAP and SIR scales. The second comprised more detailed behavioral assessments of word recognition in quiet and in noise. These evaluations were conducted with 50 participants who successfully completed the full speech assessment battery.

Clear neurophysiological associations emerged from this analysis. Intracochlear eCAEPs recorded from apical electrode #1 revealed strong negative correlations between the latencies of the P1 and N1 components and speech recognition scores in both noise (*r* = −0.75 and −0.63, respectively) and quiet (*r* = −0.63 and −0.54, respectively). In contrast, P2 latency did not show a significant relationship with speech performance. Amplitude measures of P1 and N1 were also positively and significantly associated with performance in SiN (*r* = 0.34, *r* = 0.53, respectively). These results confirm that latency and amplitude of cortical responses are key predictors of speech perception, with latency effects most pronounced for SiN.

Global auditory function measures (CAP and SIR) were also significantly and positively associated with speech recognition in both quiet and noisy conditions (e.g., CAP with speech-in-noise, *r* = 0.68, *p* < 0.01; SIR with speech-in-noise, *r* = 0.6, *p* < 0.01). CAP and SIR scores also correlated strongly with speech recognition in quiet (*r* = −0.67 and −0.4, respectively). These findings support the view that early cortical auditory responses-particularly P1 and N1 latency-serve as neurophysiological markers of speech processing efficiency in CI users.

Previous research examining the relationship between eCAEPs and speech outcomes has produced mixed results, likely due to methodological variability, differences in stimulus parameters, and recording techniques. For instance, Liebscher et al. (2018) reported modest correlations (*r* ≈ 0.33) between scalp-recorded N1–P2 amplitudes and speech recognition in adult CI users. More recently, Tavora-Vieira and Ffoulkes (2023) found weak associations between P1/P2 latency and speech recognition in quiet, though stronger correlations emerged for speech-in-noise when responses were aggregated across electrodes. In pediatric populations, Xiong et al. (2022) observed negative correlations between P1 latency and auditory outcomes, while Saravanan et al. (2024) reported moderate positive correlations between P1–N1 amplitude and speech performance in quiet, but not latency. The stronger correlations observed in the present study may be attributed to several neurophysiological and methodological advantages. First, intracochlear recordings offer a higher signal-to-noise ratio than traditional scalp EEG, minimizing contamination from non-cortical sources such as ocular or muscular artifacts. Second, the proximity of CI electrodes to neural generators along the ascending auditory pathway-including the primary auditory cortex enables the capture of temporally and spatially precise evoked potentials. This anatomical and functional alignment enhances sensitivity to cortical dynamics, which is essential for speech decoding. Third, short time-locked synchronization between stimulation and recording reduces temporal jitter and improves reproducibility across individuals, particularly for early latency components like P1 and N1, which reflect initial cortical detection and discrimination of auditory stimuli.

Further supporting this interpretation, the intracochlear eCAEPs in this study were elicited by a brief electrical stimulus delivered to a single apical electrode. While stimulation was localized, electrical spread likely activated neighboring electrodes and recruited a broader range of cochlear regions. The apical region primarily conveys low-frequency information, which is essential for prosodic features and vowel discrimination (Giraud and Lee, 2007; De Groote et al., 2024). Low frequencies also play a crucial role in speech envelope tracking, a cortical process implicated in speech comprehension and neural entrainment (Di Liberto et al., 2015). Thus, early and accurate cortical responses to apical stimulation may serve as a proxy for the fidelity of low-frequency temporal processing-an ability closely tied to speech-in-noise perception. The superiority of apical electrical stimulation in the cochlear implant for evoking enhanced and more distinct neural responses was further by direct intra cochlear auditory recordings obtained from an epilepsy patient (Nourski et al., 2013). Notably, speech recognition in noise showed stronger correlations with eCAEP latencies than recognition in quiet. This finding supports the hypothesis that noisy environments impose greater demands on the auditory system, requiring enhanced cortical engagement for speech parsing, attentional control, and auditory stream segregation (Obleser and Kayser, 2019). The ability of the auditory cortex to rapidly and accurately track incoming electrical signals-as reflected by shorter P1 and N1 latencies-may therefore represent a critical neurophysiological foundation for successful speech perception in complex listening environments.

This study demonstrated that age at cochlear implantation is the strongest predictor of auditory cortical plasticity, as reflected in both the latency and amplitude of the P1 component. Earlier implantation was associated with shorter P1 latencies and larger amplitudes, indicating more efficient and timely cortical auditory processing. This neural advantage translated into functional outcomes: individuals implanted very early in life-particularly before the age of one-achieved significantly better speech-in-noise perception compared to those implanted after the age of one, and especially those implanted after the age of 14. This relationship was clearly visualized through a box plot (Figure 10) which illustrated the distribution of speech-in-noise scores across four implantation age groups. The plot revealed a progressive decline in median performance and interquartile range with increasing age at implantation. Statistically significant differences were observed between the earliest group ( ≤ 1 year) and both the 3–8 years group and the 14–43 years group. These findings reinforce the importance of early auditory stimulation for both cortical development and real-world speech understanding in challenging listening environments. These observations further underscore the role of developmental neuroplasticity in shaping central auditory processing. In young children, the auditory system is more adaptable and capable of experience-dependent reorganization (Kral and Sharma, 2012; Ponton et al., 2000). As such, early implantation facilitates the formation of temporally precise cortical representations of speech, supporting better outcomes even under adverse listening conditions. In contrast, adults with late implantation often experience degraded or inefficient auditory encoding due to prolonged auditory deprivation, cross-modal reorganization, and reduced cortical synchrony (Lyness et al., 2013; Collignon et al., 2011; Joly et al., 2021). These maladaptive changes are reflected in prolonged cortical latencies and poorer speech performance in noise.

Caution is needed when interpreting these associations in children with additional neurodevelopmental conditions. Although intracochlear eCAEPs provide a direct and objective measure of auditory cortical activation, they cannot fully capture the complex interplay between sensory, cognitive, and linguistic systems. For instance, children with global developmental delay (GDD) or autism spectrum disorder (ASD) may exhibit age-appropriate eCAEP latencies despite significant challenges in language acquisition and auditory integration (Orekhova et al., 2014; Roberts et al., 2010). Similarly, children with auditory neuropathy spectrum disorder (ANSD) may show typical cortical responses if synaptic transmission is intact (Saki et al., 2021), yet their behavioral outcomes vary depending on brainstem synchrony and cognitive capacity. These observations highlight the need for a neurodevelopmental perspective when interpreting cortical evoked potentials in complex clinical profiles.

Our additional analysis demonstrated that LP1 latency improves prediction of speech-in-noise performance beyond age at implantation (Δ*R*^2^ = +0.133), emphasizing the unique clinical value of eCAEP. Unlike age, which is a historical factor, eCAEP offers an objective, real-time measure of cortical auditory processing. This is particularly important for identifying individual variability that age alone cannot explain, such as inconsistent device use, transition to sign language, or atypical neuroplasticity. These findings support the integration of eCAEP into clinical protocols for personalized rehabilitation and underscore its potential as a biomarker in longitudinal studies of auditory brain development.

### Limitations and future directions

While the present study provides novel and clinically relevant insights into the neural correlations of auditory and speech outcomes in CI users through intracochlear eCAEP recordings, several limitations should be acknowledged-particularly in relation to the complexity of auditory cortical processing and neurodevelopment.

First, although the sample size was moderate for a neurophysiological study, it did not allow for robust subgroup analyses across specific etiologies (e.g., congenital vs. acquired deafness), neurodevelopmental comorbidities, or varying durations of auditory deprivation. Given the heterogeneity of CI users, particularly in pediatric populations, future studies should aim to recruit larger and more homogeneous cohorts that enable stratification by clinical and neurological profiles. This is especially important when attempting to disentangle peripheral auditory function from central processing factors and broader neurocognitive development. Furthermore, the precise severity of auditory deprivation experienced of congenital/prelingually deafened subjects, despite the use of hearing aids, warrant investigation.

Second, the study employed a cross-sectional design, limiting the ability to infer causal relationships between eCAEP characteristics and long-term speech-language outcomes. Longitudinal studies incorporating repeated intracochlear eCAEP recordings-alongside developmental assessments-are essential for understanding the trajectory of cortical auditory maturation and its role in speech perception, especially under varying conditions of neuroplasticity. This approach may also help identify sensitive periods for intervention and inform individualized rehabilitation strategies.

Third, the interpretation of eCAEPs in children with comorbid neurological or neurodevelopmental disorders (e.g., global developmental delay, autism spectrum disorder, cerebral palsy) remains challenging. While intracochlear eCAEPs offer an objective measure of auditory cortical responsiveness, they do not capture the full scope of neural processing required for effective communication, including attention, executive function, and language integration. In such populations, normal-appearing cortical responses may coexist with impaired auditory comprehension, likely due to disrupted functional connectivity or atypical top-down modulation. Therefore, future research should incorporate multimodal neuroimaging (e.g., fMRI, MEG, diffusion MRI) to map the broader neural architecture underlying auditory processing and its interaction with language networks.

Another technical limitation concerns the ability to simultaneously record cortical responses to both acoustic and electric stimulation. In unilateral CI users with residual hearing in the contralateral ear-so-called bimodal users-it is possible to deliver acoustic stimulation to the hearing ear while recording cortical responses from the implanted ear (Attias et al., 2022). This approach allows direct comparison between acoustic and electric processing within the same individual, providing valuable insights into the neural mechanisms underlying auditory perception. In contrast, in unilateral CI users with bilateral profound hearing loss, ipsilateral recording from the implant side is challenging due to the presence of substantial electrical artifacts generated by the implant's stimulation. These artifacts interfere with the reliable measurement of true neural activity, obscuring cortical responses and complicating signal analysis. Therefore, there is a critical need to develop advanced artifact suppression techniques that will enable accurate ipsilateral cortical recordings using eCAEP in this population. Such advancements would significantly expand research and clinical capabilities for neurophysiological assessment in cochlear implant users, allowing a more comprehensive understanding of auditory processing in this group.

Finally, the use of a single apical electrode for stimulation, while methodologically controlled, limits generalizability across the cochlear frequency map. Although apical stimulation engages low-frequency pathways crucial for speech envelope processing, it remains unclear whether similar cortical dynamics would be observed with basal stimulation targeting high-frequency regions. Future studies should systematically vary electrode position and stimulation parameters to assess spatial tuning of cortical responses and their relation to speech perception across different phonemic ranges. Although regression analysis confirmed that chronological age does not significantly influence cortical latencies once age at implantation is considered, the possibility of residual confounding cannot be fully excluded. Future studies with larger and more balanced cohorts are needed to disentangle the relative contributions of auditory experience, age at implantation, and functional performance measures such as CAP.

## Conclusion

This study demonstrates that intracochlear electrically evoked cortical auditory evoked potentials (eCAEPs) are reliable and clinically applicable markers of auditory cortical function in bilateral cochlear implant (CI) users. The strong correlation between P1/N1 latencies and speech perception-particularly in noise-underscores their relevance as neurophysiological indicators of cortical auditory processing efficiency. These findings highlight the critical role of early implantation in promoting typical cortical development and suggest that eCAEPs may serve as objective biomarkers of auditory brain maturity.

It is important to note that the primary conclusions of this study apply to prelingually deafened participants, whose outcomes are most strongly influenced by early auditory experience. Cases involving postlingually implanted individuals with congenital or prelingual hearing loss were not analyzed as separate groups but were part of the main dataset. Due to their distinct auditory background, the interpretation of their outcomes remains preliminary and warrants further investigation.

Importantly, this is the first demonstration that intracochlear eCAEP recordings provide neuroscience with a unique research tool for directly studying brain processes associated with cochlear implant function. The technical advantages of this approach-proximity to neural generators, high signal-to-noise ratio, elimination of external recording equipment, and brief testing time-make it both feasible and informative. Incorporating eCAEPs into routine CI follow-up could enable objective monitoring of cortical maturation and guide individualized rehabilitation strategies, particularly in pediatric populations. These measures may help identify children at risk for delayed auditory development and optimize intervention timing. Future studies should explore longitudinal changes in eCAEPs, their predictive value for language outcomes, and their integration with multimodal neuroimaging to better understand cortical plasticity and cross-modal reorganization in CI users.

## Data Availability

The raw data supporting the conclusions of this article will be made available by the authors, without undue reservation.

## References

[B1] Aldag N. Büchner A. Lenarz T. Nogueira W. (2022). Towards decoding selective attention through cochlear implant electrodes as sensors in subjects with contralateral acoustic hearing. J. Neural Eng. 19:016023. doi: 10.1088/1741-2552/ac4de6 35062007

[B2] Allen C. Nikolopoulos T. P. Dyar D. O'Donoghue G. M. (2001). Reliability of a rating scale for measuring speech intelligibility after pediatric cochlear implantation. Otol. Neurotol. 22, 631–633. doi: 10.1097/00129492-200109000-00012 11568670

[B3] Anderson S. Parbery-Clark A. White-Schwoch T. Kraus N. (2012). Aging affects neural precision of speech encoding. J. Neurosci. 32, 14156–14164. doi: 10.1523/JNEUROSCI.2176-12.2012 23055485 PMC3488287

[B4] Archbold S. Lutman M. E. Nikolopoulos T. (1998). Categories of auditory performance: inter-user reliability. Br. J. Audiol. 32, 7–12. doi: 10.3109/03005364000000045 9643302

[B5] Atilgan A. Cesur S. Çiprut A. (2023). A longitudinal study of cortical auditory maturation and implications of the short inter-implant delay in children with bilateral sequential cochlear implants. Int. J. Pediatr. Otorhinolaryngol. 166:111472. doi: 10.1016/j.ijporl.2023.111472 36739687

[B6] Attias J. Greenstein T. Peled M. Ulanovski D. Wohlgelernter J. Raveh E. (2017). Auditory performance and electrical stimulation measures in cochlear implant recipients with auditory neuropathy compared with severe to profound sensorineural hearing loss. Ear Hear. 38, 184–193. doi: 10.1097/AUD.0000000000000384 28225734

[B7] Attias J. HabibAllah S. Tarigoppula V. S. A. Glick H. Chen C. Kanthaiah K. . (2022). Cortical auditory evoked potentials recorded directly through the cochlear implant in cochlear implant recipients: a feasibility study. Ear Hear. 43, 1426–1436. doi: 10.1097/AUD.0000000000001212 35245922

[B8] Bell-Souder D. Chen C. Spahr A. Sharma A. (2024). Validation of direct recording of electrically evoked cortical auditory evoked potentials through a cochlear implant system. Sci. Rep. 14:28366. doi: 10.1038/s41598-024-79528-3 39551893 PMC11570646

[B9] Bernhard N. Gauger U. Romo Ventura E. Uecker F. C. Olze H. Knopke S. . (2021). Duration of deafness impacts auditory performance after cochlear implantation: a meta-analysis. Laryngosc. Investig. Otolaryngol. 6, 291–301. doi: 10.1002/lio2.528 33869761 PMC8035957

[B10] Bertoli S. Probst R. Bodmer D. (2011). Late auditory evoked potentials in elderly long-term hearing-aid users with unilateral or bilateral fittings. Hear. Res. 280, 58–69. doi: 10.1016/j.heares.2011.04.013 21569828

[B11] Blamey P. Artieres F. Başkent D. Bergeron F. Beynon A. Burke E. . (2013). Factors affecting auditory performance of postlinguistically deaf adults using cochlear implants: an update with 2251 patients. Audiol. Neurotol. 18, 36–47. doi: 10.1159/000343189 23095305

[B12] Bogdanov C. Goulios H. Mulders W. H. A. M. Tavora-Vieira D. (2024). Investigating the effect of cochlear implant usage metrics on cortical auditory-evoked potential responses in adult recipients post-implantation. Front. Neurosci. 18:1453274. doi: 10.3389/fnins.2024.1453274 39640296 PMC11619141

[B13] Callejón-Leblic M. A. Barrios-Romero M. M. Kontides A. Sánchez-Gómez S. Beynon A. J. (2022). Electrically evoked auditory cortical responses elicited from individually fitted stimulation parameters in cochlear implant users. Int. J. Audiol. 62, 650–658. doi: 10.1080/14992027.2022.2062578 35477333

[B14] Chen Y. Li J. Li Y. Zhang Y. (2024). Features of the speech processing network in post- and prelingually deaf cochlear implant users. Cereb. Cortex 34:bhad417. doi: 10.1093/cercor/bhad417 38163443

[B15] Collignon O. Vandewalle G. Voss P. Albouy G. Charbonneau G. Lassonde M. . (2011). Functional specialization for auditory–spatial processing in the occipital cortex of congenitally blind humans. Proc. Nat. Acad. Sci. 108, 4435–4440. doi: 10.1073/pnas.1013928108 21368198 PMC3060256

[B16] Davis H. Mast T. Yoshie N. Zerlin S. (1966). The slow response of the human cortex to auditory stimuli: recovery process. Electroencephalogr. Clin. Neurophysiol. 21, 105–113. doi: 10.1016/0013-4694(66)90118-0 4162003

[B17] De Groote E. Carlyon R. P. Deeks J. M. Macherey O. (2024). Effects of selective stimulation of apical electrodes on temporal pitch perception by cochlear implant recipients. J. Acoust. Soc. Am. 156, 2060–2076. doi: 10.1121/10.0029023 39345135 PMC11444735

[B18] Deniz H. (2025). Auditory performance and cortical plasticity following late sequential cochlear implantation. Audiol. Neurotol. 1–14. doi: 10.1159/000547204 40639365

[B19] Dettman S. J. Pinder D. Briggs R. J. S. Dowell R. C. Leigh J. R. (2007). Communication development in children who receive the cochlear implant younger than 12 months: risks versus benefits. Ear Hear. 28, 11S−18S. doi: 10.1097/AUD.0b013e31803153f8 17496638

[B20] Di Liberto G. M. O'Sullivan J. A. Lalor E. C. (2015). Low-frequency cortical entrainment to speech reflects phoneme-level processing. Curr. Biol. 25, 2457–2465. doi: 10.1016/j.cub.2015.08.030 26412129

[B21] Edwards E. Soltani M. Kim W. Dalal S. S. Nagarajan S. S. Berger M. S. . (2009). Comparison of time-frequency responses and the event-related potential to auditory speech stimuli in human cortex. J. Neurophysiol. 102, 377–386. doi: 10.1152/jn.90954.2008 19439673 PMC2712274

[B22] Ghiselli S. Gheller F. Trevisi P. Rampazzo P. Ermani M. Martini A. (2016). The impact of age and duration of cochlear implant in a congenital deaf population: an ERP study. J. Biomed. Sci. Eng. 9, 384–392. doi: 10.4236/jbise.2016.98033

[B23] Gifford R. H. Shallop J. K. Peterson A. M. (2008). Speech recognition materials and ceiling effects: considerations for cochlear implant programs. Audiol. Neurotol. 13, 193–205. doi: 10.1159/000113510 18212519

[B24] Giraud A.-L. Lee H.-J. (2007). Predicting cochlear implant outcome from brain organization in the deaf. Restor. Neurol. Neurosci. 25, 381–390. doi: 10.3233/RNN-2007-253420 17943013

[B25] Glennon E. Svirsky M. A. Froemke R. C. (2020). Auditory cortical plasticity in cochlear implant users. Curr. Opin. Neurobiol. 60, 108–114. doi: 10.1016/j.conb.2019.11.003 31864104 PMC7002179

[B26] Groenen P. A. Beynon A. J. Snik A. F. van den Broek P. (2001). Speech-evoked cortical potentials recognition in cochlear implant users and speech. Scand. Audiol. 30, 31–40. doi: 10.1080/010503901750069554 11330917

[B27] Guo Q. Li Y. Fu X. Liu H. Chen J. Meng C. . (2016). The relationship between cortical auditory evoked potentials (CAEPs) and speech perception in children with Nurotron ^®^ cochlear implants during four years of follow-up. Int. J. Pediatr. Otorhinolaryngol. 85, 170–177. doi: 10.1016/j.ijporl.2016.03.035 27240518

[B28] HabibAllah S. Chen C. Attias J. (2025). Direct recording of electrically evoked cortical potentials from cochlear implants demonstrates feasibility and clinical relevance in pediatric users. Sci. Rep. 15:22644. doi: 10.1038/s41598-025-06652-z 40595118 PMC12217970

[B29] He S. Grose J. H. Teagle H. F. Woodard J. Park L. R. Hatch D. R. . (2015). Acoustically evoked auditory change complex in children with auditory neuropathy spectrum disorder. Ear Hear. 36, 289–301. doi: 10.1097/AUD.0000000000000119 25422994 PMC4409935

[B30] Holden L. K. Finley C. C. Firszt J. B. Holden T. A. Brenner C. Potts L. G. . (2013). Factors affecting open-set word recognition in adults with cochlear implants. Ear Hear. 34, 342–360. doi: 10.1097/AUD.0b013e3182741aa7 23348845 PMC3636188

[B31] Jafari Z. Fitzpatrick E. M. Schramm D. R. Rouillon I. Koravand A. (2024). Predictors of cochlear implant outcomes in pediatric auditory neuropathy: a matched case-control study. PLoS ONE 19:e0304316. doi: 10.1371/journal.pone.0304316 38809896 PMC11135674

[B32] Jeong S. W. Chung S. H. Kim L.-S. (2018). P1 cortical auditory evoked potential in children with unilateral or bilateral cochlear implants; implication for the timing of second cochlear implantation. Eur. Arch. Otorhinolaryngol. 275, 1759–1765. doi: 10.1007/s00405-018-5021-5 29855691

[B33] Joly C. A. Reynard P. Hermann R. Seldran F. Gallego S. Idriss S. . (2021). Intra-cochlear current spread correlates with speech perception in experienced adult cochlear implant users. J. Clin. Med. 10:5819. doi: 10.3390/jcm10245819 34945115 PMC8709369

[B34] Kelly A. S. Purdy S. C. Thorne P. R. (2005). Electrophysiological and speech perception measures of auditory processing in experienced adult cochlear implant users. Clin. Neurophysiol. 116, 1235–1246. doi: 10.1016/j.clinph.2005.02.011 15978485

[B35] Kraaijenga V. J. C. Derksen T. C. Stegeman I. Smit A. L. (2017). The effect of side of implantation on unilateral cochlear implant performance in patients with prelingual and postlingual sensorineural hearing loss: a systematic review. Clin. Neurophysiol. 43, 440–449. doi: 10.1111/coa.12988 28944603

[B36] Kral A. Heid S. Hubka P. Tillein J. (2013). Unilateral hearing during development: Hemispheric specificity in plastic reorganizations. Front. Syst. Neurosci. 7:93. doi: 10.3389/fnsys.2013.00093 24348345 PMC3841817

[B37] Kral A. Sharma A. (2012). Developmental neuroplasticity after cochlear implantation. Trends Neurosci. 35, 111–122. doi: 10.1016/j.tins.2011.09.004 22104561 PMC3561718

[B38] Kral A. Tillein J. (2006). Brain plasticity under cochlear implant stimulation. Adv. Otorhinolaryngol. 64, 89–108. doi: 10.1159/000094647 16891838

[B39] Lee D. S. Lee J. S. Oh S. H. Kim S. K. Kim J. W. Chung J. K. . (2001). Cross-modal plasticity and cochlear implants. Nature 409, 149–150. doi: 10.1038/35051653 11196628

[B40] Lee H.-J. Smieja D. Polonenko M. J. Cushing S. L. Papsin B. C. Gordon K. A. (2020). Consistent and chronic cochlear implant use partially reverses cortical effects of single-sided deafness in children. Sci. Rep. 10:78371. doi: 10.1038/s41598-020-78371-6 33298987 PMC7726152

[B41] Liebscher T. Alberter K. Hoppe U. (2018). Cortical auditory evoked potentials in cochlear implant listeners via single electrode stimulation in relation to speech perception. Int. J. Audiol. 57, 939–946. doi: 10.1080/14992027.2018.1514469 30295156

[B42] Lyness C. R. Woll B. Campbell R. Cardin V. (2013). How does visual language affect crossmodal plasticity and cochlear implant success? Neurosci. Biobehav. Rev. 37, 2621–2630. doi: 10.1016/j.neubiorev.2013.08.011 23999083 PMC3989033

[B43] Martin B. A. Boothroyd A. (1999). Cortical, auditory, evoked potentials in response to changes of spectrum and amplitude. J. Acoust. Soc. Am. 107, 2155–2161. doi: 10.1121/1.428556 10790041

[B44] Morlet T. Valania J. Walter C. Morini G. O'Reilly R. C. Parkes W. . (2023). Cortical auditory evoked potential testing in children with auditory neuropathy spectrum disorder. Am. J. Audiol. 33, 171–182. doi: 10.1044/2023_AJA-23-00051 38048283

[B45] Nassar A. A. M. Hassan D. M. Rahman T. T. A. Younis A. (2020). Speech evoked potentials in cochlear implant recipients with auditory neuropathy spectrum disorder. Hear. Balance Commun. 19, 133–143. doi: 10.1080/21695717.2020.1836578

[B46] Niparko J. K. (2010). Spoken language development in children following cochlear implantation. JAMA 303:1498. doi: 10.1001/jama.2010.451 20407059 PMC3073449

[B47] Nourski K. V. Etler C. P. Brugge J. F. Oya H. Kawasaki H. Reale R. A. . (2013). Direct recordings from the auditory cortex in a cochlear implant user. J. Assoc. Res. Otolaryngol. 14, 435–450. doi: 10.1007/s10162-013-0382-3 23519390 PMC3642273

[B48] Obleser J. Kayser C. (2019). Neural entrainment and attentional selection in the listening brain. Trends Cogn. Sci. 23, 913–926. doi: 10.1016/j.tics.2019.08.004 31606386

[B49] Orekhova E. V. Elsabbagh M. Jones E. J. Dawson G. Charman T. Johnson M. H. . (2014). EEG hyper-connectivity in high-risk infants is associated with later autism. J. Neurodev. Disord. 6:40. doi: 10.1186/1866-1955-6-40 25400705 PMC4232695

[B50] Ponton C. W. Eggermont J. J. Kwong B. Don M. (2000). Maturation of human central auditory system activity: evidence from multi-channel evoked potentials. Clin. Neurophysiol. 111, 220–236. doi: 10.1016/S1388-2457(99)00236-9 10680557

[B51] Purcell P. L. Deep N. L. Waltzman S. B. Roland J. T. Jr. Cushing S. L. Papsin B. C. . (2021). Cochlear implantation in infants: why and how. Trends Hear. 25:23312165211031751. doi: 10.1177/23312165211031751 34281434 PMC8295935

[B52] Roberts T. P. Khan S. Y. Rey M. Monroe J. F. Cannon K. Blaskey L. . (2010). MEG detection of delayed auditory evoked responses in autism spectrum disorders: towards an imaging biomarker for autism. Autism Res. 3, 8–18. doi: 10.1002/aur.111 20063319 PMC3099241

[B53] Ross B. Tremblay K. (2009). Stimulus experience modifies auditory neuromagnetic responses in young and older listeners. Hear. Res. 248, 48–59. doi: 10.1016/j.heares.2008.11.012 19110047 PMC2668103

[B54] Sahwan M. Abdelsamad Y. Alasfoor F. Alfayez F. Binkhamis G. Nichani J. (2024). Cochlear implantation in children with auditory neuropathy spectrum disorder: an updated systematic review. Eur. Arch. Otorhinolaryngol. 281, 1149–1162. doi: 10.1007/s00405-023-08194-4 37638998

[B55] Saki N. Nikakhlagh S. Moridi B. Karimi M. Aghayi A. Bayat A. (2021). Cortical auditory plasticity following cochlear implantation in children with auditory neuropathy spectrum disorder: a prospective study. Otol. Neurotol. 42, e1227–e1233. doi: 10.1097/MAO.0000000000003257 34172662

[B56] Saravanan P. Devi N. Geetha C. (2024). Electrically evoked late latency response using single electrode stimulation and its relation to speech perception among paediatric cochlear implant users. Front. Hum. Neurosci. 18:1441854. doi: 10.3389/fnhum.2024.1441854 39345947 PMC11427271

[B57] Schwab B. (2015). Duration of deafness impacts auditory performance after cochlear implantation: a meta-analysis. Int. J. Otolaryngol. Article ID 528. 33869761 10.1002/lio2.528 PMC8035957

[B58] Shader M. J. Luke R. McKay C. M. (2022). Contralateral dominance to speech in the adult auditory cortex immediately after cochlear implantation. iScience 25:104737. doi: 10.1016/j.isci.2022.104737 35938045 PMC9352526

[B59] Sharma A. Cormier K. Grigsby J. (2025). Effect of supplemental language therapy on cortical neuroplasticity and language outcomes in children with hearing loss. Brain Sci. 15:119. doi: 10.3390/brainsci15020119 40002452 PMC11853721

[B60] Sharma A. Dorman M. F. Kral A. (2005). The influence of a sensitive period on central auditory development in children with unilateral and bilateral cochlear implants. Hear. Res. 203, 134–143. doi: 10.1016/j.heares.2004.12.010 15855038

[B61] Sharma A. Dorman M. F. Spahr A. J. (2002). A sensitive period for the development of the central auditory system in children with cochlear implants: implications for age of implantation. Ear Hear. 23, 532–539. doi: 10.1097/00003446-200212000-00004 12476090

[B62] Sharma A. Nash A. A. Dorman M. (2009). Cortical development, plasticity and re-organization in children with cochlear implants. J. Commun. Disord. 42, 272–279. doi: 10.1016/j.jcomdis.2009.03.003 19380150 PMC2696307

[B63] Silva L. A. Couto M. I. Magliaro F. C. Tsuji R. K. Bento R. F. de Carvalho A. C. . (2013). Long latency auditory evoked potentials in children with cochlear implants: systematic review. CoDAS 25, 595–600. doi: 10.1590/S2317-17822013.05000009 24626971

[B64] Silva L. A. Couto M. I. Magliaro F. C. Tsuji R. K. Bento R. F. de Carvalho A. C. . (2017). Cortical maturation in children with cochlear implants: correlation between electrophysiological and behavioral measurement. PLoS ONE 12:e0171177. doi: 10.1371/journal.pone.0171177 28151961 PMC5289550

[B65] Steinmetzger K. Rupp A. (2024). The auditory P2 is influenced by pitch changes but not pitch strength and consists of two separate subcomponents. Imaging Neurosci. 2, 1–16. doi: 10.1162/imag_a_00160 40800308 PMC12247569

[B66] Tavora-Vieira D. Ffoulkes E. (2023). Direct elicitation of cortical auditory evoked potentials by electrical stimulation and their use to verify the most comfortable level of stimulation in cochlear implant users. Audiol. Neurotol. 28, 294–307. doi: 10.1159/000529797 36958296 PMC10407833

[B67] Távora-Vieira D. Mandruzzato G. Polak M. Truong B. Stutley A. (2021). Comparative analysis of cortical auditory evoked potential in cochlear implant users. Ear Hear. 42, 1755–1769. doi: 10.1097/AUD.0000000000001075 34172688

[B68] Távora-Vieira D. Wedekind A. Ffoulkes E. Voola M. Marino R. (2022). Cortical auditory evoked potential in cochlear implant users: an objective method to improve speech perception. PLoS ONE 17:e0274643. doi: 10.1371/journal.pone.0274643 36206248 PMC9543874

[B69] Wang X. Lin Z. Guo Y. Liu Y. Zhou X. Bai J. . (2023). Correlation between cortical auditory evoked potential and auditory speech performance in children with cochlear implants. Int. J. Pediatr. Otorhinolaryngol. 172:111687. doi: 10.1016/j.ijporl.2023.111687 37515869

[B70] Wilson B. S. Dorman M. F. (2008). Cochlear implants: a remarkable past and a brilliant future. Hear. Res. 242, 3–21. doi: 10.1016/j.heares.2008.06.005 18616994 PMC3707130

[B71] Xiong S. Jiang L. Wang Y. Pan T. Ma F. (2022). The role of the P1 latency in auditory and speech performance evaluation in cochlear implanted children. Neural Plast. 2022, 1–10. doi: 10.1155/2022/6894794 35422857 PMC9005287

